# Supplementary material for: Protocol for an international multicenter randomized controlled trial assessing treatment success and safety of peroral endoscopic myotomy vs endoscopic balloon dilation for the treatment of achalasia in children
Source: PLoS One. 2023 Oct 5;18(10):e0286880. doi: 10.1371/journal.pone.0286880 (PMC10553306; doi:10.1371/journal.pone.0286880)
Supplement: S1 Checklist — (DOCX) [file pone.0286880.s001.docx]

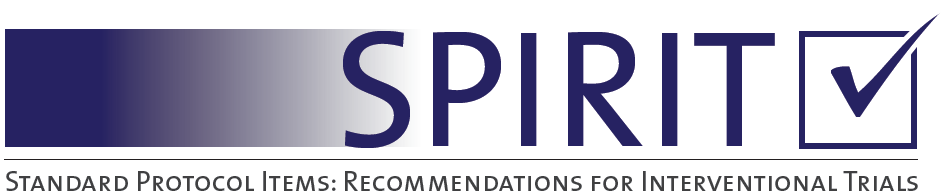


SPIRIT 2013 Checklist: Recommended items to address in a clinical trial protocol and related documents*

| Section/item | ItemNo | Description |
| --- | --- | --- |
| **Administrative information** | | |
| Title | 1 | Protocol for an international multicenter randomized controlled trial assessing treatment success and safety of peroral endoscopic myotomy vs endoscopic balloon dilation for the treatment of achalasia in children. |
| Trial registration | 2a | ISRCTN registry. Study registration number: ISRCTN74448884 |
|  | 2b | See table at the end of the document |
| Protocol version | 3 | Original: 04-2021  Amendement: 05-2022 |
| Funding | 4 | No external funding |
| Roles and responsibilities | 5a | PF, AB, B.B., MB, MvW designed the first draft of this protocol and all authors contributed substantially to the conception of the study.  JHvdL gave statistical- and methodological advice. The protocol was drafted by MS, MvL and CM. The protocol was refined by all other authors. All authors have read and approved the final protocol. |
|  | 5b | Emma Children’s Hospital  Meibergdreef 9 1105 AZ Amsterdam  020-5669111 |
|  | 5c | Not applicable |
|  | 5d | **Principal Investigator and subinvestigator** Preparation of protocol and revisions Preparation of and CRFs  Publication of study reports  **Steering committee (SC)** MvW, MvL, CM Agreement of final protocol. Recruitment of patients  Reviewing progress of study and if necessary agreeing changes to the protocol and/or investigators brochure to facilitate the smooth running of the study.  **Trial Management Committee (TMC)** MvW, MB, MvL, CM Study planning Provide annual risk report  Responsible for trial master file Audit of 6 monthly feedback forms and decide when site visit to occur. Assistance with international review, board/independent ethics committee applications Data verification Randomisation |
| Introduction |  |  |
| Background and rationale | 6a | Achalasia is a rare neurodegenerative esophageal motility disorder characterized by incomplete lower esophageal sphincter (LES) relaxation, increased LES tone and absence of esophageal peristalsis. |
|  | 6b | Achalasia requires invasive treatment in all patients. Conventional treatment options include endoscopic balloon dilation (EBD) and laparoscopic Heller’s myotomy (LHM). Recently, a less invasive endoscopic therapy has been developed; Peroral Endoscopic Myotomy (POEM). POEM integrates the theoretical advantages of both EBD and LHM (no skin incisions, less pain, short hospital stay, less blood loss and a durable myotomy). |
| Objectives | 7 | To compare efficacy and safety of POEM vs. EBD as primary treatment for achalasia in children |
| Trial design | 8 | Multi-center, and center-stratified block-randomized controlled trial. Blocks of 2,4 and 6 are applied. |
| Methods: Participants, interventions, and outcomes | | |
| Study setting | 9 | The data will be collected in an academic hospital in the Netherlands. |
| Eligibility criteria | 10 | Inclusion criteria: Eckardt score > 3; presence of a HRM pattern consistent with achalasia type I or II according to the latest Chicago classification (CC) criteria and age 7 up to and including 17 years at the time of screening visit.  Exclusion criteria: Achalasia type III; Previous surgical or endoscopic achalasia treatment; Previous surgery of the upper gastrointestinal tract; Known coagulopathy; Known Liver cirrhosis and/or esophageal varices; Known LA grade ≥B esophagitis; Known Barrett’s esophagus; Known pregnancy at time of treatment; Stricture of the esophagus; Known presence of malignant or premalignant esophageal lesions; Hiatal hernia > 1cm based on HRM measurement ; Extensive, tortuous dilatation (>7cm luminal diameter, S shape) of the esophagus; Barium esophagram suggestive of other pathologies.  All (pediatric) gastro-enterologist performing POEM or EBD have performed at least 15 prior to the study. |
| Interventions | 11a | See page 9 of the manuscript |
|  | 11b | Not applicable |
|  | 11c | Not applicable |
|  | 11d | Not applicable |
| Outcomes | 12 | Primary: the need for any retreatment. secondary: Achalasia symptoms (Eckardt score); Health-related- and disease specific QoL.  LA grade (EGD) HRM and 24 hour pH-impedance (pH-MII) measurement parameters  Stasis on contrast esophagram 1 minute after ingestion of barium  Procedure times Complications (any unwanted events that arise following treatment and/or that are secondary to the treatment) severe and mild. |
| Participant timeline | 13 | See figure 1 of the manuscript |
| Sample size | 14 | 126 |
| Recruitment | 15 | Achalasia will be diagnosed in participating centre, patient will immediately receive information on standard treatment and the trial. |
| **Methods: Assignment of interventions (for controlled trials)** | | |
| Allocation: |  |  |
| Sequence generation | 16a | Computer randomized |
| Allocation concealment mechanism | 16b | Not applicable |
| Implementation | 16c | Principle investigator and sub-investigator are responsible for randomisation. |
| Blinding (masking) | 17a | Not applicable |
|  | 17b | Not applicable |
| **Methods: Data collection, management, and analysis** | | |
| Data collection methods | 18a | The Eckhardt score, PedsQl, PedsQL-GI, reflux disease questionnaire, achalasia specific QoL questionnaire, barium esophagram, HRM, pH-MII and EGD will be used to answer primary and secondary objectives. |
|  | 18b | Telephone calls will be made instead of hospital visits to promote participant retention. |
| Data management | 19 | Data will be collected and stored in a digital database (Castor). Original documents will be stored on site. |
| Statistical methods | 20a | See page 13 and 14 of manuscript |
|  | 20b | Not applicable |
|  | 20c | Not applicable |
| **Methods: Monitoring** | | |
| Data monitoring | 21a | A monitor will be appointed in all participating centres. |
|  | 21b | An interim report will be made after 30 participants reached T=12 months and send to the DSMB. |
| Harms | 22 | All adverse events (severe and mild) will be collected during the trial. |
| Auditing | 23 | Not applicable |
| Ethics and dissemination | | |
| Research ethics approval | 24 | This study has been approved by the Medical Ethics Research Committee of the AMC hospital (NL68967.018.20).  An independent DSMB consisting of an epidemiologist, a pediatric gastroenterologist and an adult gastroenterologist will evaluate safety of participants after 50 participants have reached 1 year follow up. |
| Protocol amendments | 25 | Not applicable |
| Consent or assent | 26a | The PI and subinvestigator will obtain informed consent from the patient and their parents/ caregivers (when aged < 16) |
|  | 26b | Not applicable |
| Confidentiality | 27 | Personal information will be collected at baseline and stored in the ISSF file at location and the patients electronic patient file. Data will be shared anonymously unless patient gives permission to share information. |
| Declaration of interests | 28 | No conflict of interests are present. |
| Access to data | 29 | The PI and a subinvestigator from the Emma Children’s hospital will have access to the final dataset. |
| Ancillary and post-trial care | 30 | Participants will remain under care of the Emma Children’s hospital after trial.  An insurance is present to compensate those who undergo harm as a result of the trial. |
| Dissemination policy | 31a | The results of this RCT will be presented at national and international conferences and published in an international medical journal |
|  | 31b | The manuscript will list all participating professionals. |
|  | 31c | Not applicable |
| Appendices |  |  |
| Informed consent materials | 32 | **ICF see appendix** |
| Biological specimens | 33 | Not applicable |

*It is strongly recommended that this checklist be read in conjunction with the SPIRIT 2013 Explanation & Elaboration for important clarification on the items. Amendments to the protocol should be tracked and dated. The SPIRIT checklist is copyrighted by the SPIRIT Group under the Creative Commons “[Attribution-NonCommercial-NoDerivs 3.0 Unported](http://www.creativecommons.org/licenses/by-nc-nd/3.0/)” license.

2B:

Appendix: PIF

**Subject information letter for participation in medical-scientific research**

**PEDPOEM**

*Per oral endoscopic myotomy (POEM) versus endoscopic balloon dilation (EBD) as treatment for pediatric achalasia.*

**Introduction**

Dear sir/madam,

You received this letter because your child was diagnosed with achalasia for which he/she will be treated shortly.

We ask your permission for your child to participate in research. Participation is voluntarily. To participate is a written consent necessary. Before you decide if you want your child to participate, you will receive information about the study. Read the information carefully and ask the researcher any questions you may have. You can also talk about it with your partner, friends or family members.

1. **General information**

This research is conducted by the Emma Children’s Hospital, Amsterdam UMC and will be executed by doctors in multiple hospitals in different countries.

This study will require a total of 82 children and teenagers. As achalasia is a rare disease, children from different countries will participate. The medical-ethical committee in the AMC has approved this study.

1. **Purpose of the study**

The main purpose of this study is to investigate which treatment is the best and safest option for children with achalasia. To answer this question, we will compare the results of the endoscopic stretching with a balloon treatment to the endoscopic cutting of the esophageal muscles. At the end of this study, the results will be written down and published to ensure that other doctors also know what the best and safest treatment option is for children with achalasia. The published data will not be traceable to your child, see page 7 for more information on your child’s privacy.

1. **Background of the study**

Achalasia is a rare disease of the esophageal nerves. The muscles are no longer properly controlled by the affected nerves. This prevents peristaltic movements in the esophagus and prevents the lower esophageal muscle from relaxing properly. A constriction occurs at the transition from esophagus to the stomach, so that food and drink cannot pass from the esophagus to the stomach. Children with achalasia have difficulty eating and drinking and can experience pain. They may also suffer from vomiting and unwanted weight loss. The symptom onset is usually between the ages of 7 and 13 years old. But the first symptoms can also occur at a younger or older age. Untreated achalasia can lead to distention of the esophagus and aggravation of the symptoms.

The current treatment options for achalasia include an endoscopic or surgical treatment (abdominal surgery).

With **Endoscopic Balloon Dilation (EBD)**, a bendable hose with a camera at the end (endoscope), will be introduced through the mouth into the stomach. A small balloon is attached to the scope and will be inflated at the junction from the esophagus to the stomach. This will stretch the junction muscles, causing food to pass easier from esophagus to stomach. Children are under anesthesia during this procedure and will not notice anything of it. Usually this takes place in day treatment: which means your child can go home on the same day. With this treatment there is a 1 in 70 chance (1.5%) of a rupture of the esophageal wall, which needs a small operation to close. Children often need multiple balloon dilations to ensure disappearance of symptoms.

There has been a new treatment for achalasia for a few years now: **Per Oral Endoscopic Myotomy (POEM)**. With the POEM treatment, an endoscope is introduced through the mouth into the stomach. At the transition from esophagus to the stomach, the endoscope cuts a part of the esophageal muscles, to that food and drinks will pass into the stomach easier. This elevates the constriction. Children are under anesthetics during this procedure and will therefore not notice anything of it. POEM is currently not an option as first treatment of achalasia in children outside the research setting. POEM is sometimes performed in children with persisting symptoms despite balloon treatment (EBD). If this is the case with your child, POEM could be a treatment option. This procedure is performed in children multiple times and the results are good. The risk and type of complications that can occur, like a esophageal wall perforation, is about the same in the three procedures.

1. **What does participation entail?**
   1. *Before the treatment*

Your child can participate in the study when he/ she is diagnosed with achalasia and is not yet treated for it. If you decide to let your child participate in the study, he/ she will be randomized between the two treatment options: endoscopic stretching of the esophageal muscle (standard of care) or endoscopic cutting of the esophageal muscles (the new treatment). Both treatments will be performed by a pediatric gastroenterologist. Your child will remain treated with his/her own doctor.

Before treatment, some blood will be drawn to rule out other diseases which may cause a problem when treating achalasia. This will also be done if your child does not participate in the study.

If your child participates, you will be asked to complete a number of questionnaires together with your child. Filling out these questionnaires will take about 30 minutes.

- 1. *The treatment*

**If your child is allocated fort he endoscopic balloon dilation**

If your child is allocated for **endoscopic balloon dilation (EBD)**, he/she will receive the standard of care treatment, children with achalasia in our hospital receive this treatment first, even if they do not participate in this study. This treatment is done twice, with an 2-4 week interval. In the first session, the muscles is stretched 3 cm. The second time, the muscle will be stretched 3,5 cm. If the symptoms reoccur within 6 months, you will receive a third dilation to 3,5 cm. 48 hours before treatment, your child is only allowed to drink clear fluids and 8 hours before treatment he/she is not allowed to eat or drink anything. This is to make sure your esophagus is completely empty. This is also necessary if you do not participate in the study. The treatment is performed in day care: your child can go home on the same day. The first year after treatment, your child will receive acid suppressive medication.

**If your child is allocated for Per Oral Endoscopic Myotomy (POEM)**

If your child is allocated for **Per Oral Endoscopic** **Myotomy (POEM)**, he/she will receive the new treatment for children with achalasia. In this hospital, several children and adults received this treatment, however we do not know if POEM is better than the current standard treatment for children. Your child is under anesthesia during the treatment and will therefore not notice anything of it. To prevent infections, your child will receive antibiotics before the treatment. 48 hours before treatment, your child is only allowed to drink clear fluids and 8 hours before treatment he/she is not allowed to eat or drink anything. Your child will stay in hospital for 1-2 days after the treatment. The first year after treatment, your child will receive acid suppressive medication. The first two weeks after the treatment your child can only eat soft and grinded food, to make sure the esophagus can heal.

After both treatments, your child will stay in follow-up by us in this study for one year and occasionally we will ask you to complete some questionnaires. We will also do some tests one year after the initial treatment. See appendix C for an overview of the study visits.

*4.3. After treatment*

**Which extra tests will your child receive if he/she participates in the study?**

After the treatment will your child receive extra tests, which are otherwise only performed when your child’s symptoms reoccur. By comparing the results from the two tests, we can see which treatment is most appropriate for children with achalasia. If your child participates in the study, your child will have a barium swallow and esophageal motility test (manometry), 12 months after treatment. If your child does not participate, these tests are only performed when your child’s symptoms reoccur. Your child will also have an endoscopy and pH-impedance study 12 months after the first treatment. These tests are also done if your child does not participate in the study. In addition to the tests, we will asks you to complete questionnaires at various times before and after treatment, which include questions on symptoms due to achalasia and your child general health and wellbeing. The chart below shows which examinations are extra for this study.

Chart of all standard of care tests and study related tests:

**Before treatment Randomization After treatment**

**Standard of care**

- Endoscopy after 12 months and every 3 years thereafter

- pH impedance test after 12 months

- Questionnaire on achalasia symptoms

- At reoccurrence of symptoms:

- Esophageal motility test

- Barium swallow

- pH-impedance test and/or

- Endoscopy

**Standard of care**

- Esophageal motility test

- Barium swallow

- Questionnaire on achalasia symptoms

- Drawing some blood

**Extra**

- Quality of life questionnaire

- Reflux disease questionnaire

**Treatment:**

**Endoscopic**

**dilation**

or

**Endoscopic cutting**

**Extra**

- Endoscopy 12 months after treatment

- Barium swallow 12 months after treatment

- Esophageal motility test 12 months after treatment

- Quality of life and reflux disease questionnaires at 3, 6 and 12 months.

- Monthly weight at home during the first year

**What happens if your child does not participate in the study?**If you decide that you do not want your child to participate in the study, he/she will receive standard of care. The current standard treatment is the endoscopic balloon dilation.

**Visits and measurements**During the 12 months of the study, it is necessary to have three standard visits at the hospital. In appendix C you can find an overview of the expectations each visit. If your child does not participate in the study, he/ she will have a similar number of follow-up visits.

1. **What do we expect from your child?**

In order to let the study run smoothly, it is important that your child adheres to the following expectations:

The expectations we have for your child are that:

- He/ she shows up for the follow-up visits at the hospital
- He/ she weighs him/herself every month at home at the scale which is provided by the hospital.
- He/ she completes the questionnaires at the appropriate times

It is important that you contact the researcher when:

- Your child starts on new medication. Even if these are homeopathic, natural medication, vitamins or medication from the drug store.
- Your child is admitted or treated in hospital.
- Your child suddenly develops health issues.
- Your child does not want to participate anymore.
- Your child’s contact details change.

1. **Possible complications or side effects**

**Are there any risks or side effects in participating?**

Both balloon dilation and POEM are treatments that have potential risks. The risk for a complication and the type of complications are similar for both treatments. With both treatments you are at risk for a rupture of the esophageal wall or a bleed. There is also a risk of infection. These complications can be severe and usually need direct treatment. They may lead to an elongation of the hospital stay or a new procedure.

Reflux as a result of achalasia treatment
With all achalasia treatments, the intention is to weaken the lower esophageal muscle by stretching or cutting it. This makes it easier for food and drinks to pass through to the stomach. Unfortunately, this also makes it easier for gastric acid to flow back into the esophagus, this is called reflux. If this happens too often, it is called reflux disease. Exactly how often reflux disease occurs in children treated for achalasia has not yet been well researched. It seems that reflux disease may occur more often after POEM (1 in 6 to 7 children) compared to balloon dilation (1 in 10 children). Usually this type of reflux disease is well manageable with medication. As a precaution, everyone who participates in this study will receive this medicine. 12 months after treatment, an endoscopy and a pH impedance study are performed to determine whether the medication is (still) necessary.

1. **Potential advantages and disadvantages**

It is important to take the possible advantages and disadvantages into account when deciding whether you want your child to participate in the study.

**Inconveniences of the tests**

- Motility test and pH impedance tests: the positioning of the catheters might be slightly unpleasant. The throat can be slightly irritated. A bleeding nose occurs very rarely. This resolves by itself.
- Endoscopy; a bleed of the esophagus and a tear in the esophageal wall may occur. In addition, an infection can occur.
- Barium Swallow: the radiation your child is exposed to during a barium swallow is 2 mSV. The study requires one extra barium swallow. This means that your child is exposed to a total of 2 mSV extra radiation. This is seen as a medium risk. The ‘normal’ radiation everyone is exposed to in the Netherlands is about 2.5 mSV annually.

Participating does also mean that:

- - you and your child spend some time completing questionnaires. It takes about 30 minutes to complete the questionnaires each time
  - your child needs to weigh him/herself every month at home
  - you have agreements that your child must keep.

All previous points are discussed in paragraph 4, 5 and 6.

*What are the disadvantages of tests using radiation?*A barium swallow uses radiation. The radiation your child is exposed to during a barium swallow is 2 mSV. By way of comparison: the ‘normal’ radiation everyone in the Netherlands is exposed to every year is about 2 mSv. It is not a problem for your child to be exposed to radiation for a medical reason or treatment.

- Is your child exposed to radiation more often? Talk to the researcher whether it is wise for your child to participate.
- The radiation used during the barium swallow may be a potential health risk for your child. However, this is a small risk. We do advise not to let your child participate in another study using radiation.

**Potential advantages**

There are no personal advantages for your child to participate. Both treatments elevate symptoms. Risk of complications is also comparable for both treatments. If your child is enrolled in the study and receives POEM, a potential benefit to your child may be that he/she receive a therapy that is not yet available outside a research setting. In adults, POEM is a very effective treatment (Ponds et al. JAMA 2019). Your child’s participation helps us investigating the best and safest treatment option for children with achalasia.

1. **Your child’s resistance**

It is possible that your child shows resistance (is uncooperative) during the study. The researcher must stop the study immediately. It is difficult to exactly describe resistance. What resistance looks like for your child will be discussed before every test. The researcher will adhere to the Code of Conduct for Underage Patients.

1. **If you do not want your child to participate or want to stop with the study**

You decide if you want your child to participate. Participation is voluntarily.
If you do not want your child to participate, he/she will receive standard of care.

If you do want your child to participate, you can always change your mind. If you want to stop before the initial treatment was given, your child will receive standard care (EBD). It is also possible to stop after initial treatment. The follow-up will resume as normal. You do not have to tell why your child stops the study. You do have to inform the researcher. The data collected thus far will be used for the study.

If new information concerning the study arises, the researcher will inform you and ask whether you want your child to continue in the study.

1. **End of the study**

Participation ends when:

- All study visits are completed
- You choose to stop with the study
- The researcher decides it is best for your child to stop
- The Emma Children’s Hospital, the government or the approving medical-ethical commission decides to terminate the study.

The entire study is done when all participants had treatment and completes all questionnaires. The researcher will inform you on the first results of the study after data-analysis. This takes place about 4 years after the initial start of the study. The study is not done then. You will receive the final results about 6 years after the start of the study.

**The use and storage of your child’s data**

This study requires the collection, usage and storage of your child’s personal data. This concerns data such as name, address, date of birth and information about your child’s health. The collection, usage and storage of data is necessary to answer the questions this study asks and to publish the results. We ask for your permission to use your child’s data.

**Confidentiality of your data**

To protect your child’s privacy, all data will be coded. Name and other information that can directly identify your child will be omitted. Only with a key to the code will the data be traceable to your child. The key is safely stored in the local research institute. Data send to the initial researcher consist of the coded data only, not the name or other identifiable data. Even in reports and publications about the research, the data cannot be traced back to your child.

**Access to your child’s data**

A small proportion of researchers at the local research site has access to all data. Even the decoded data. This is necessary to be able to check if the research is conducted properly and trustworthy. People who will have access to your child’s data: a committee that keeps an eye on the safety of the research, a monitor who works for the researcher, national and international authorities, for example the Health and Youth Care inspectorate. They keep the data secret. We ask for your permission to give these people access.

**Retention period data and body materials**

The data will be stored for 20 years at the research site.

**Storage and usage of data for another study**After this study, your child’s data may also be important for other scientific research in the field of achalasia. The data will be stored for that purpose. You can indicate on the informed consent form whether or not you agree with this. If you do not want this, your child can still participate in the current study.

**Information on unexpected findings**

During the study, something may accidentally be found that is not important for the study, but is important for your child. If it is important for his/ her health, you will be informed by your doctor. You can talk to your general practitioner or medical specialist about a solution. You also give permission for this.

**Withdrawal of consent**

You can always withdraw your consent. This applies for the current study and the storage and usage for future research. The data collected thus far will be used up to the moment of withdrawal of consent.

**More information on your rights when processing data**

General information on your rights when processing your child’s personal data can be found on the Data Protection Authorities website.

With questions or complaints concerning data processing, we advise to contact the study site first. You can always contact the data privacy officer (appendix A) or the Data Protection Authority.

See appendix A for contact details of the Emma Children’s Hospital and the principal investigators.

**Study registration**

A description of this clinical study is on www.toetsingonline.nl. This website does not contain traceable information. It does contain a summary of the results. You have access to this website. You can find this study when searching for ‘PEDPOEM’.

1. **Participants insurance**

An insurance is taken out for every participant. The insurance covers any harm as a result of participation in the study. Not all harm is covered. You can find more information on the insurance and its exceptions in Appendix B. It also states who you can report the harm to.

1. **Informing the general practitioner**

We always inform the general practitioner about the study participation. This is important for his/her safety. If you do not agree, your child cannot participate. Neither can your child participate if he/she does not have a general practitioner.

1. **No compensation for participation**

You will not get paid for your child to participate.

1. **Do you have any questions?**

If you have any questions, you can contact the research team. For independent advice about participating, you can contact the independent doctor. He knows a lot about the study, but is not directly associated with this study.
If you have any complaints about the study, you can talk to the researcher or your doctor. If prefer not to do this, you can contact the complaints officer. All details can be found in **appendix A**: contact details.

1. **Signing the informed consent form**

After a maximum of 2 weeks to think, you are asked to decide on your child’s participation in this study. If you give permission, we ask you to sign the corresponding informed consent form. Your written consent indicates that you understand the information and consent to your child’s participation in the study. Both you and the research will receive a signed version of this consent form.

Thank you for your attention.

1. **Appendices with this information**

A. Contact details Emma Children’s Hospital, Amsterdam UMC, locatie AMC

B. Information about the insurance

C. Chart stating the study visits

D. Informed consent form parents/ guardian

**Appendix A: contact details Emma Children’s Hospital Amsterdam UMC, locatie AMC**

Principal Investigator: Dr. M.P. van Wijk, pediatric gastroenterologist, Emma Children’s Hospital, Amsterdam UMC. Email: m.vanwijk@amsterdamumc.nl; phone number: 020-5662906

Executive investigator: Marinde van Lennep, research doctor, pediatric gastroenterology, Emma Children’s Hospital, Amsterdam UMC. E-mail: pedpoem@amsterdamumc.nl; phone number: 06-50063838

Independent doctor: Dr. B. Koot, pediatric gastroenterologist, Emma Children’s Hospital, Amsterdam UMC. Email: b.koot@amsterdamumc.nl

Complaints: patient information and complaints: 020 - 566 3355 or by email klachten@amsterdamumc.nl

More information on your child’s rights: data privacy officer Amsterdam UMC, AMC: privacy@amsterdamumc.nl

**Appendix B: information about the insurance**

Amsterdam UMC has taken out insurance for everyone participating in this study. The insurance covers harm as a result of the study. This applies to harm during the study up to 4 years after your participation. It has to be reported to the insurance within 4 years.

The insurance does not cover all damage. Below you will find what harm is not covered. These provision are set out in the ‘Compulsory insurance for medical research involving humans 2015’. This decision is in the law of the Dutch government (<https://wetten.overheid.nl>).

When any harm is done you can contact the insurance company:

Study insurance company:

Name: Centramed B.A.

Address: Postbus 7374, 2701 AJ Zoetermeer

Phone number: 070 301 70 70

E-mail: [info@centramed.nl](mailto:info@centramed.nl)

Number: 624.528.303

The insurance covers €650.000 per participant and 5.000.000 for the entire study, and 7.500.000 per year for all studies conducted in the Amsterdam UMC.

The following damage is **not** covered:

- damage from a risk you were informed about in the written information. This does not apply when the risk is greater than expected or when the risk was very small;
- any health damage which would have arisen when you did not participate in the study;
- damage caused by not (fully) following directions or instructions;
- damage to your descendants, as a result of a negative effect of the research on you or your descendants
- damage as a result of existing treatment methods in research into existing treatment methods.

**Appendix C – Overview study visits**

|  | **0 months** (before treatment) | **3 months** | **6 months** | **1 year** |
| --- | --- | --- | --- | --- |
| **Questionnaires on:**   - Achalasia symptoms - Quality of life - Reflux symptoms | X | X | X | X |
| **Weight**  All children will receive a scale and have to weigh themselves every months at home for the first year after treatment. | X | X | X | X |
| **Height**  Your child’s height will be measured every 3 months. If you have a phone appointment, this can be done at the general practitioner. | X | X | X | X |
| **Barium swallow**  Esophageal passage x-ray | X | - | - | X |
| **High resolution manometry**  Esophageal motility test | X | - | - | X |
| **Endoscopy**  Camera that looks at the inside of the esophagus | X  (during treatment) | - | - | X |
| **pH impedance test**  measures the acidity of the esophagus during 24H. | - | - | - | X |

**Appendix D: informed consent form parent/ guardian**

**PEDPOEM**

I have been asked to give permission for my child/ below mentioned person to participate in medical research:

Name participant (child):

Date of birth: __ / __ / __

- I have read the information letter for parents/ guardians. I was able to ask questions. My questions were sufficiently answered. I have had enough time to decide on my child’s participation.
- I know participation is voluntary. I also know I can decide to stop the study at any moment. I do not have to give an explanation.
- I give permission to inform the general practitioner about the study.
- I give permission to request information on my child’s weight and height from the general practitioner/ specialist.
- I am aware that for the purpose of checking the study certain people have access to my child’s details. These people are listed in this information letter. I give permission for these people to have access.
- I give permission to inform the general practitioner/ current specialist about unexpected findings which might be of importance to my child’s health.
- I □ **do**

□ **do not**
give permission to store my child’s data for future research on achalasia.

- I □ **do**

□ **do not**give permission to approach my child for a follow-up study.

- I consent that my child participates in this study.

Name parent/guardian**:

signature: Date: __ / __ / __

Name parent/guardian**:

Signature: Date: __ / __ / __

-----------------------------------------------------------------------------------------------------------------

I declare that I fully informed above mentioned person on the study.

If information becomes known during the study that could influence the consent of the parent or guardian, I will inform him/her in a timely manner.

Name researcher (or its representative):

Signature: Date: __ / __ / __

-----------------------------------------------------------------------------------------------------------------

Additional information is provided by:

Name:

Role:

Signature Date: __ / __ / __

-----------------------------------------------------------------------------------------------------------------

* Strike out what does not apply.

** If the child is under the age of 16 years of age, the parent(s) with parental authority or the guardian will sign this form. Children aged 12-15 who can decide for themselves (are competent), need to sign a form themselves.

*The parent/ guardian receives the information letter and the signed informed consent form.*

| **Data category** | **Information** |
| --- | --- |
| Primary registry and trial identifying number | ISCRTN - 74448884 |
| Date of registration in primary registry | 18-02-2022 |
| Secondary identifying numbers | EudraCT: 2022-000474-26 |
| Source(s) of monetary or material support | Emma Children’s Hospital |
| Primary sponsor | Emma Children’s Hospital |
| Secondary sponsor(s) | Not applicable |
| Contact for public queries | M.P. van Wijk, MD, PhD  020-5662906  m.vanwijk@amsterdamumc.nl |
| Contact for scientific queries | M.P. van Wijk, MD, PhD  020-5662906  m.vanwijk@amsterdamumc.nl |
| Public title | PerOral Endoscopic Myotomy (POEM) versus Endoscopic Balloon Dilatation (EBD) for the treatment of achalasia in children (PedPOEM). |
| Scientific title | PerOral Endoscopic Myotomy (POEM) versus Endoscopic Balloon Dilatation (EBD) for the treatment of achalasia in children (PedPOEM). |
| Countries of recruitment | The Netherlands |
| Health condition(s) or problem(s) studied | Achlasia |
| Intervention(s) | PerOral Endoscopic Myotomy |
| Key inclusion and exclusion criteria | Inclusion criteria: Eckardt score > 3; presence of a HRM pattern consistent with achalasia type I or II according to the latest Chicago classification (CC) criteria and age 7 up to and including 17 years at the time of screening visit.  Exclusion criteria: Achalasia type III; Previous surgical or endoscopic achalasia treatment; Previous surgery of the upper gastrointestinal tract; Known coagulopathy; Known Liver cirrhosis and/or esophageal varices; Known LA grade ≥B esophagitis; Known Barrett’s esophagus; Known pregnancy at time of treatment; Stricture of the esophagus; Known presence of malignant or premalignant esophageal lesions; Hiatal hernia > 1cm based on HRM measurement ; Extensive, tortuous dilatation (>7cm luminal diameter, S shape) of the esophagus; Barium esophagram suggestive of other pathologies. |
| Study type | Interventional  Allocation: randomized  Primary purpose: treatment |
| Date of first enrolment | 09-02-2022 |
| Recruitment status | Recruiting |
| Primary outcome(s) | Primary outcome measure is the need for any retreatment. |
| Key secondary outcomes | Achalasia symptoms (Eckardt score); Health-related- and disease specific QoL.  LA grade (EGD) HRM and 24 hour pH-impedance (pH-MII) measurement parameters  Stasis on contrast esophagram 1 minute after ingestion of barium  Procedure times  Complications (any unwanted events that arise following treatment and/or that are secondary to the treatment) severe and mild. |
