# Supplementary material for: Protocol for an international multicenter randomized controlled trial assessing treatment success and safety of peroral endoscopic myotomy vs endoscopic balloon dilation for the treatment of achalasia in children
Source: PLoS One. 2023 Oct 5;18(10):e0286880. doi: 10.1371/journal.pone.0286880 (PMC10553306; doi:10.1371/journal.pone.0286880)
Supplement: S1 File — (PDF) [file pone.0286880.s002.pdf]

Peroral endoscopic myotomy (POEM) vs endoscopic balloon dilation (EBD) for the treatment of achalasia in children

PROTOCOL TITLE

'Peroral endoscopic myotomy (POEM) vs endoscopic balloon dilation (EBD) for the treatment of achalasia in children'

**Protocol ID**	**PEDPOEM_01**
**Short title**	**Endoscopic myotomy vs endoscopic dilation for achalasia in children**
**Coordinating investigator/project leader**	***Dr. Michiel P. van Wijk (m.vanwijk@amsterdamumc.nl)***
**Principal investigator(s) (in Dutch: hoofdonderzoeker/ uitvoerder)**	***Emma Kinderziekenhuis, Amsterdam UMC, location AMC, The Netherlands:***<br>***Dr. Michiel van Wijk (m.vanwijk@amsterdamumc.nl)***
<Multicenter research: per site>	
Emma Childrens Hospital, Amsterdam UMC, location AMC, The Netherlands	*Prof. Dr. M.A. Benninga (m.a.benninga@amsterdamumc.nl)*<br>*Prof. Dr. A.J. Bredenoord (a.j.bredenoord@amsterdamumc.nl)*<br>*Prof. Dr. P. Fockens (p.fockens@amsterdamumc.nl)*
Emma Childrens Hospital, Amsterdam UMC, location VUmc, The Netherlands.	*Dr. Michiel van Wijk (m.vanwijk@amsterdamumc.nl)*

For other participating centers, please refer to the seperate ***List of paticipating centers****.*	
**Sponsor (in Dutch: verrichter/opdrachtgever)**	***Afdeling Kindermaagdarmleverziekten, Emma Kinderziekenhuis, Academic Medical Center***
**Subsidising party**	***NA***
**Laboratory sites <*if applicable*>**	***NA***
**Pharmacy <*if applicable*>**	***NA***

PROTOCOL SIGNATURE SHEET

**Name**	**Signature**	**Date**
**Head of Department:**	Prof. Dr. W.B. de Vries, head of department of pediatrics	
**Principal Investigator:**	Prof Dr. M.A. Benninga, principal investigator.	

TABLE OF CONTENTS

1. INTRODUCTION AND RATIONALE 15
2. OBJECTIVES 18
3. STUDY DESIGN 19
4. STUDY POPULATION 20
4.1 Population (base) 20
4.2 Inclusion criteria 20
4.3 Exclusion criteria 20
4.4 Sample size calculation 21
5. TREATMENT OF SUBJECTS 21
5.1 Investigational treatment 191
5.2 Retreatment in case of treatment failure 22
6. METHODS 22
6.1 Study parameters/endpoints 22
6.1.1 Main study parameter/endpoint 22
6.1.2 Secondary study parameters/endpoints (if applicable) 22
6.2 Randomization, blinding and treatment allocation 23
6.3 Study procedures 24
6.4 Withdrawal of individual subjects 34
6.4.1 Specific criteria for withdrawal (if applicable) 34
6.5 Follow-up of subjects withdrawn from treatment 34
6.6 Premature termination of the study 34
7. SAFETY REPORTING 34
7.1 Temporary halt for reasons of subject safety 35
7.2 AEs, SAEs and SUSARs 35
7.2.1 Adverse events (AEs) 35
7.2.2 Serious adverse events (SAEs) 35
7.4 Follow-up of adverse events 38

7.5 [Data Safety Monitoring Board (DSMB) / Safety Committee] 38
8. STATISTICAL ANALYSIS 38
9. ETHICAL CONSIDERATIONS 39
9.1 Regulation statement 39
9.2 Recruitment and consent 39
9.3 Objection by minors or incapacitated subjects (if applicable) 40
9.4 Benefits and risks assessment, group relatedness 40
9.5 Compensation for injury 42
9.6 Incentives (if applicable) 42
10. ADMINISTRATIVE ASPECTS, MONITORING AND PUBLICATION 42
10.1 Handling and storage of data and documents 42
10.2 Amendments 43
10.3 Annual progress report 43
10.4 Temporary halt and (prematurely) end of study report 43
10.5 Public disclosure and publication policy 44
11. REFERENCES 45

LIST OF ABBREVIATIONS AND RELEVANT DEFINITIONS

AE	Adverse Event
AMC	Academic Medical Center
BMI	Body Mass Index
CC	Chicago Classification
DSMB	Data Safety Monitoring Board
DSQL	Disease Specific Quality of Life
EBD	Esophageal Balloon Dilatation
EoE	Eosinophilic Esophagitis
EGD	Esophago-gastro-duodenoscopy
EGJ	Esophageal Gastric Junction
GER	Gastro-esophageal Reflux
GERD	Gastro-Esophageal Reflux Disease
HRM	High Resolution Manometry
HRIM	High Resolution Impedance Manometry
HRQoL	Health Related QoL
ICF	Informed Consent Form
IRB	Institutional Review Board
IRP4	Integrated Resting Pressure 4s
LA	Los Angeles classification (endoscopic classification system for reflux esophagitis)
LES	Lower Esophageal Sphincter
LHM	Laparoscopic Heller's Myotomy
MREC	Medical research ethics committee;
PedsQL	Pediatric Quality of Life Inventory
POEM	Peroral Endoscopic Myotomy
pH-MII	pH-impedance
PPI	Proton Pump Inhibitor
QoL	Quality of life

**RCT**	**Randomized Controlled Trial**
**RDQ**	**Reflux Disease Questionnaire**
**(S)AE**	**(Serious) Adverse Event**
**SAP**	**Symptom Association Probability**
**SI**	**Symptom Index**
**SSI**	**Symptom sensitivity index**
**Sponsor**	**The sponsor is the party that commissions the organisation or performance of the research, for example a pharmaceutical company, academic hospital, scientific organisation or investigator. A party that provides funding for a study but does not commission it is not regarded as the sponsor, but referred to as a subsidising party.**
**WMO**	**Medical scientific research involving human subjects is covered by the Medical Research Involving Human Subjects Act (WMO)**

SUMMARY

Rationale: Achalasia is a rare neurodegenerative esophageal motility disorder characterized by incomplete lower esophageal sphincter (LES) relaxation, increased LES tone and absence of esophageal peristalsis. Clinical symptoms include dysphagia, regurgitation, chest pain and weight loss.(1, 2) In children, achalasia is most commonly diagnosed after the age of 7 and has an estimated incidence of 0.1-0.18/100.000 children per year.(2, 3) Achalasia requires invasive treatment in all patients. This treatment is aimed at reducing the resistance to flow at the level of the esophagogastric junction (EGJ) by reducing LES pressure. This leads to improvement of esophageal emptying and thereby a decrease in symptoms. Conventional treatment options include endoscopic balloon dilation (EBD) and laparoscopic Heller's myotomy (LHM).(2) The downside of EBD is the frequent recurrence of disease where additional treatment is needed, while LHM carries a serious risk of complications due to surgery.(4)

Recently, a less invasive endoscopic alternative to perform a myotomy via a submucosal tunnel has been developed; Peroral Endoscopic Myotomy (POEM).(5) POEM integrates the theoretical advantages of both EBD and LHM (no skin incisions, less pain, less blood loss and yet a durable myotomy).

POEM has shown to be very effective and safe in adults on the short and long-term (up to 51 months).(6-9) Although EBM, LHM and POEM are currently performed to treat children with achalasia, randomized controlled trials (RCTs) evaluating their safety and efficacy are lacking. Several single center and uncontrolled studies with follow-up times up to 53 months, have shown that POEM is feasible in children aged 1 year up to 18 years.(10-15) These studies have shown promising results with symptomatic success rates of > 93% in the absence of severe adverse events.(15) To date only one pediatric study retrospectively compared the efficacy and safety of the two endoscopic therapies (EBD = 9 or POEM = 12 patients) in terms of treatment success (disease specific symptom Eckardt Score ≤3), need for re-treatment, complications and susceptibility to gastroesophageal reflux disease (GERD) in the longer term.(16) Treatment success of POEM and EBD was 100% vs 66.7% respectively after 12 months and 100% vs 33.3% respectively after 36 months. No serious complications were reported.(16)

Objective: To compare efficacy and safety of POEM vs. EBD as primary treatment for achalasia in children.

Study design: Multi-center, center-stratified block-randomized controlled trial in which POEM will be compared with EBD.

Study population: Treatment naïve pediatric patients (age 7-18 years) diagnosed with achalasia based on Eckardt score > 3 AND high resolution manometry (HRM) consistent with achalasia (Integrated resting pressure above upper limits of normal, with absent peristalsis or panesophageal pressurization).(17)

Intervention (if applicable): POEM vs EBD

Main study parameters/endpoints: Primary outcome measure is the need for retreatment (i.e. EBD, LHM, POEM or intra-sphincteric botox injection) due to persisting symptoms (Eckardt score > 3) *with* evidence of recurrence on barium swallow and/*or* HRM within 12 months follow-up, as assed by a blinded end-point committee (PROBE design) Need for retreatment also includes retreatment that is planned *before* 12 months follow-up, but scheduled *after* 12 months follow-up.
Secondary outcomes include:

- Achalasia symptoms defined by the Eckardt symptom score
- Health-related- and disease specific quality of life (QoL)
- Gastroesophageal reflux disease (GERD) symptoms defined by the reflux disease questionnaire
- Grade of esophagitis defined by Los Angeles (LA) grade during esophagogastroduodenoscopy (EGD)
- Degree of GERD defined by 24 hour pH-impedance (pH-MII) measurement parameters
- Esophageal and lower esophageal sphincter function defined by HRM parameters
- Procedure related complications
- Procedure times

Nature and extent of the burden and risks associated with participation, benefit and group relatedness: Achalasia requires invasive treatment in all patients. In most centers, EBD is proposed as a first step treatment in children.(18)

Participants who receive EBD in this study, will be treated similarly as newly diagnosed achalasia patients who are not included in this study. They will therefore not be exposed to additional risks. Several studies in children have reported that EBD is safe (four cohort studies, n=58, follow-up up to 10 years; four comparative studies, n=197, follow-up up to 10 years).(2, 19-25) Complications occurred rarely and included esophageal perforation (n=5/209 and 1/60 respectively), post dilation fever (n=2/10) and postoperative pain (n=1/10).

Success rates in these small, and often retrospective studies, varied widely (10% up to 90%).(26) When comparing these results in children with those from a large RCT in adult achalasia patients, results regarding safety are comparable.(27) Younger adults (≤40 years old) had significantly worse clinical success rates (Eckardt ≤3) than older patients (66% vs 92% at 5-years follow-up respectively). It is therefore necessary to evaluate achalasia therapies in the pediatric age-group to assess therapeutic success and safety in this specific patient group.

An endoscopic alternative for EBD is POEM, which is currently increasingly used in adult patients. POEM is currently not available as a first-step treatment for children in our center. In case of symptom relapse with abnormal esophagogram and/or high resolution manometry however, in our center POEM is offered as relapse treatment after failed EBD or LHM when patients prefer this therapy after extensive counselling between available options. Several children have already successfully received POEM . In this study, POEM will be the investigational therapy. The largest cohort study in children (n=44, follow-up 1 – 53 months) reported only mild intraoperative complications including retroperitoneal CO_2 (7%), capnoperitoneum (3%), and mucosal injury (1%).(14) POEM was technical feasible in 98% and had a clinical success rate (Eckardt score ≤3) of 82% at 4-year follow-up.(14) These results are in line with adult literature, where clinical success (Eckardt score ≤3) was achieved in >90% (follow-up 3 – 51 months).(9, 28) POEM was performed in more than 300 adult patients and approximately 10 pediatric patients (>7 years old) in our center and until now, no perforations have occurred. Benefits of receiving POEM over EBD may include a higher therapeutic success rate in terms of symptom relief and need for retreatment.

All therapeutic options aim to lower the LES pressure, which leads to a disrupted anti-reflux barrier function, which as a consequence may cause GERD post-achalasia therapy.

In children, only one study retrospectively assessed the prevalence of GERD post-EBD and reported one out of 12 patients (8%) with GERD two years after EBD.(21) The largest retrospective pediatric POEM study (n=44) evaluated GERD three months post-therapy. EGD was performed in 20 patients and revealed erosive esophagitis (LA Grade B) in eight patients. pH-impedance tests were performed in 13 patients of which 3 had increased esophageal acid exposure time (>7%). PPIs were ceased 1 week prior to measurement.(14)

A recently published RCT in adults comparing POEM (n=63) with EBD (n=63) performed an endoscopic evaluation of GERD at 1 and 2 years post-therapy.(9) Treatment success (defined as an Eckardt score ≤3 and the absence of severe complications or re-treatment) at 2 year follow up was significantly higher in patients who underwent POEM (92% vs 54%). At 12 months follow-up, all patients underwent an EGD off-PPI. Esophagitis was present in 41% vs 7% in patients who underwent POEM and EBD respectively. In summary, based on adult studies and limited retrospective pediatric studies, POEM seems to be more effective than EBD at the cost of more GERD. Which one of the two leads to a higher quality of life in children remains to be investigated.(9)

Although most investigations and hospital visits of study participants are considered standard achalasia care, participants need to fill in one additional questionnaire (not considered burdensome for their psychological well-being) and they have a strict follow-up scheme on a regular base which requires some extra time compared to achalasia patients that are not enrolled in this study. (see figure 1) Participants in this study do not need to visit the hospital more often.

Figure 1: scheme with standard and additional investigations and hospital visits during study period

Prior to therapy

Standard care
- HRM
- Contrast esophagram
- Eckardt score

Additional investigations
- QoL questionnaire
- Reflux disease questionnaire (RDQ)

Randomization

therapy:

EBD

vs

POEM

After therapy

Standard care
- EGD after 12 months and afterwards every 3 years
- Eckardt score every 3 months
- In case of symptom relapse:
  - HRM
  - contrast esophagogram
  - pH-MII and/or
  - EGD

Additional investigations
- EGD at T=12 months
- Contrast esophagram at T=12 months
- pH-MII at T=12 months
- HRM at T=12 months
- RDQ and QoL questionnaires at T=3, 6, and 12 months)
- Weight assessment at home on a monthly basis until T=12 months
- Eckardt score on a monthly basis until T = 12 months

1. INTRODUCTION AND RATIONALE

Background

Achalasia is a rare neurodegenerative esophageal motility disorder characterized by incomplete lower esophageal sphincter (LES) relaxation, increased LES tone and absence of esophageal peristalsis.

Symptoms include dysphagia, regurgitation, chest pain and weight loss.(2) In children, achalasia is mostly diagnosed after the age of 7 and has an estimated incidence of 0.1-0.18/100.000 children per year.(2, 3) Children with achalasia experience significant morbidities associated with this condition, occurring both pre- and post-therapy.(2, 29) If left untreated, patients will become tube-feeding dependent. In the long term, incomplete esophageal emptying as a consequence of achalasia may lead to the development of squamous cell carcinoma of the esophagus.(30, 31) Multiple relapse therapies and the impact of the swallowing problems in social situations may be what upsets children most. Children report feeling uncomfortable when eating with peers and even with family.(29) Although literature on quality of life (QoL) of pediatric achalasia is scarce, one small cross-sectional questionnaire study reports that children with achalasia have lower QoL in comparison to healthy children and children with inflammatory bowel disease.(29) One other study reported decreased QoL in adulthood in patients who had developed achalasia during childhood.(2)

When symptoms suggestive of achalasia are present, a (timed) barium swallow is generally performed which may reveal esophageal stasis and dilation above the LES. If this is present, the gold standard test for diagnosing achalasia in both children and adults, esophageal high resolution manometry (HRM) is performed. According to the Chicago Classification of esophageal motility disorders (CC), achalasia is present when the integrated LES relaxation pressure (IRP4) is above the upper limit of normal of 15mmHg combined with absent esophageal peristalsis or spasm.(27, 32)

Current treatment

Treatments of achalasia are aimed at reducing the resistance to flow at the level of the esophagogastric junction (EGJ) by reducing LES pressure. This leads to improvement of esophageal emptying and thereby a decrease in symptoms and prevention of megaesophagus at the long-term. In children, the optimal therapy of choice is still under debate.(26) A recent online-based survey amongst members of the European and North American Societies for Pediatric Gastroenterology Hepatology and Nutrition showed that treatment of pediatric achalasia varies widely amongst different centers world-wide.(18)

Endoscopic balloon dilation

Endoscopic balloon dilation (EBD) is considered the most effective *non-surgical* treatment for achalasia in adults and is offered as the first-line treatment in children in most achalasia centers.(2) However, many children with achalasia are in need of multiple relapse dilatations after initial EBD and a substantial group eventually requires surgery.(26) Adult literature has suggested that younger patients (<40 years) have a shorter clinical response to EBD, with only modest improvement in clinical response after repeat dilatations.(3) A review by our group on studies comparing EBD with laparoscopic Heller's myotomy (LHM) found relapse rates to range from 71% to 90% after EBD compared with 22% to 39% after LHM (follow-up 1 – 22 years).(2, 23-25) All these procedures carry a significant impact on QoL.(33) For a growing child, who faces a lifetime of potential repeat procedures, this is specifically relevant.

Heller's myotomy (LHM)

LHM is a surgical alternative for EBD. During LHM, the circular muscle fibers of the LES are cut. In most achalasia centers, LHM is offered as a second-step therapy after EBD therapy has failed.(34) Compared with EBD, LHM is a more invasive technique as it requires abdominal laparoscopic surgery.(2, 23, 24) Where EBD can be performed as an outpatient procedure, LHM involves a hospital stay of usually one or two nights. Although clinical success rates in children are high and up to 85% (follow-up up to 14 years), complications including perforations also often occur and are reported in up to 10% of children.(35)

<u>Investigational treatment</u>

Peroral endoscopic myotomy (POEM)

In 2009, an endoscopic means to perform the myotomy via a submucosal tunnel was developed; Peroral Endoscopic Myotomy (POEM).(5) POEM integrates the theoretical advantages of both endoscopic dilation (no skin incisions, decreased pain, and less blood loss) and LHM (durable surgical myotomy and single procedure).

POEM is nowadays widely used to treat adult achalasia patients due to its excellent outcomes and minimal risk of major adverse events.(6, 7, 27, 28) A large randomized controlled trial in adults comparing efficacy of POEM vs EBD, reported a one year success rate (Eckardt score ≤ 3) of 92.2% vs 70% respectively, in the absence of major complications for both procedures.(9) Although POEM has shown to be effective and safe in adults, only very few pediatric studies have investigated its feasibility and efficacy.(10-13, 16, 36-38) The studies available were single center and uncontrolled, but nevertheless showed promising results with symptomatic success rates of >90% at an overall follow-up of 1 to 2 years.(14, 15) The largest (retrospective) series of 44 children (mean age 14 years old) reported treatment success of 93% after one year follow-up and 83% after four years follow-up. Only one patient had mucosal injury, which could be treated with endoscopic clips during the procedure.(14)

To date, only one small pediatric study retrospectively compared the efficacy and safety of the two endoscopic therapies (EBD = 9 and POEM = 12 patients) in terms of treatment success (Eckardt Score ≤ 3), need for re-treatment, complications and susceptibility to gastroesophageal reflux disease (GERD) in the longer term. Treatment success of POEM was 100% at 12 and 36 months follow-up, whereas for EBD, treatment success was only achieved in 66.7% and 33.3% of patients at the respective time-points. None of the procedures was associated with serious complications. Two patients who were treated with POEM developed symptomatic esophagitis and were successfully treated with proton pump inhibitors (PPI).(16)

2. OBJECTIVES

Primary Objective: to compare safety and efficacy of POEM and EBD as first-line treatment for achalasia in children based on a superiority design.

Primary outcome measure: the need for retreatment (i.e. EBD, LHM, POEM or intra-sphincteric botox injection performed *or* scheduled) due to recurrent or persisting symptoms (Eckardt score > 3) *with* evidence of recurrence on barium swallow *and/or* HRM within 12 months after initial treatment (see page 21 and 22 for further explanation), as assed by a blinded end-point committee (PROBE design)

Secondary Objective(s):

To compare treatment success and safety, the following secondary outcomes are measured at the applicable time points during follow-up (i.e. at 3, 6 and 12 months after initial treatment, see page 26 for follow up schedule, see page 29 for accepted variations from the follow-up schedule):

- Achalasia symptoms defined by the Eckardt symptom score
- Health-related- and disease specific QoL
- Gastroesophageal reflux (GER) symptoms defined by the reflux disease questionnaire
- Grade of esophagitis defined by LA grade during esophagogastroduodenoscopy (EGD)
- Degree of GERD defined by 24 hour pH-impedance (pH-MII) measurement parameters
- Esophageal and LES function defined by HRM parameters
- Procedure related complications
- Procedure times

For definition of treatment failure and initial treatments see page 22.

<u>Table 1:</u> Study parameters of HRM, pH-MII and EGD

Measurement	Outcomes
**High resolution manometry**	Achalasia subtype I / II according to CC (17)
	LES resting pressure (mmHg)
	LES integrative relaxation pressure (mmHg)
	Distal contractile integral (mmHg·s·cm)
	Distal latency (S)
**pH-impedance**	Acid exposure time (% pH < 4)<br>- total<br>- upright position<br>- supine position
	Number of reflux episodes<br>- upright vs supine

	- acid (pH < 4)/ weakly acidic (pH 4 – 7) / alkalic (pH >7)
	Symptom indices<br>- Symptom Index<br>- Symptom sensitivity index<br>- Symptom association probability
	Esophageal stasis defined by bolus presence time
**Esophagogastroduodenoscopy**	- macroscopic abnormalities<br>- LA esophagitis classification<br><u>Additional parameters at T=12 months:</u><br>- microscopic signs of inflammation<br>- microscopic count of eosinophils

3. STUDY DESIGN

This is a multicenter center-stratified block-randomized controlled clinical trial in which POEM will be compared with EBD as first-step treatment for children with achalasia. The primary endpoint will be measured at 12 months after initial treatment. Treatment outcome will be evaluated by a blinded end-point committee (PROBE design). (56)

4. STUDY POPULATION

4.1 Population (base)

Treatment naïve pediatric patients (age from 7 up to 17 years inclusive) diagnosed with achalasia type I or II, based on Eckardt score > 3 AND HRM pattern consistent with achalasia.

4.2 Inclusion criteria

In order to be eligible to participate in this study, a subject must meet all of the following criteria:

- Eckardt score > 3
- presence of a HRM pattern consistent with achalasia type I or II according to the latest Chicago classification (CC) criteria
- Age 7 years up to 17 years inclusive

4.3 Exclusion criteria

A potential subject who meets any of the following criteria will be excluded from participation in this study:

- Achalasia type III (this extremely rare subtype in children that is accompanied by esophageal spasm and associated complaints has a worse prognosis compared to type I and II. Literature and expert-opinion based recommendations suggest to treat type III with POEM. Randomizing these patients to EBD with a high a priori risk of relapses is therefore considered unethical)
- Previous surgical or endoscopical achalasia treatment
- Previous surgery of the upper gastrointestinal tract
- Known coagulopathy
- Known Liver cirrhosis and/or esophageal varices
- Known LA grade ≥B esophagitis
- Known Barrett's esophagus
- Known pregnancy at time of treatment
- Stricture of the esophagus
- Known presence of malignant or premalignant esophageal lesions
- Hiatal hernia > 1cm based on HRM measurement
- Extensive, tortuous dilatation (>7cm luminal diameter, S shape) of the esophagus
- Barium esophagram suggestive of other pathologies

4.4 Sample size calculation

A recent retrospective review of 21 pediatric achalasia patients, of which 12 received POEM and 9 EBD, reported treatment success (Eckardt score ≤ 3) at 12, 24 and 36 months follow-up to be 100% for POEM at all follow up moments and 66.7% vs 44.4% vs 33.3% at 12, 24 and 36 months follow-up for EBD patients respectively.(16) However, in this study no repeated EBD-treatment was allowed in case of clinical recurrence. In adults 76% of patients treated according to our protocol had clinical success after 12 months.(54) Age is known to be a factor in predicting

success-rates of EBD with worse outcome in younger patients.

Taken all this together, we assumed a success rates of 72.5% for EBD and 92.5% for POEM in children after one year, we estimated that with 56 patients in each group, the study would have 80% power to detect a significant difference in success rate between EBD and POEM with a two-sided alpha level of 0.05. To cope with an estimated 10% loss to follow-up, we aim to enroll a total number of 126 patients (63 patients in each group).It is assumed that patient enrollment will be accomplished within 5 years, after approval from all local institutional review boards (IRBs).

5 TREATMENT OF SUBJECTS

5.1 Investigational product/treatment

In this study, the investigational treatment is POEM. This procedure is performed as described by Inoue et al.(5) For a detailed description of the procedure see under Methods, Section 4.

'Initial therapy' is defined as:

- POEM: a single POEM procedure
- EBD: a series of two EBD sessions, scheduled two weeks apart (see page 29 for allowed variations from the study schedule).
    - In case of *persisting* symptoms as evaluated 7 days after the second EBD session, a third EBD session (35mm in children <12 years; 40mm in children ≥12 years old) will be performed 2-4 weeks after the second. Persisting symptoms in this regards are defined as an Eckardt score>2. Note that a lower score is considered positive (as compared to diagnosis / relapse) due to the fact that the evaluation of weight loss within a week after treatment is considered inaccurate.
    - In case of *relapse* (defined as recurrent symptoms (Eckardt score >3) and evidence of active disease on barium esophagram and/or HRM), a second series of two EBD 2-4 weeks apart will be performed as part of the initial therapy. For this second series a 30 & 35mm balloon is used in children <12 years and a 35 & 40mm balloon is used in children ≥12 years old).

5.2 Retreatment in case of treatment failure

Retreatment is indicated in case of treatment failure, defined as:

- Symptom recurrence: Eckardt score > 3 (see page 31) AND
- Barium esophagram with esophageal stasis at T= 1 minute, and/or HRM (performed if barium esophagram is inconclusive) with IRP4 above upper limits of normal (15mmHg) (39, 40).

These tests are part of standard clinical care and are performed in all achalasia patients who have persisting- or recurrent symptoms after treatment.

Retreatment (LHM, POEM, EBD or intra-sphincteric botox injection) will be offered at the discretion of local preferences and/or the attending physician.

6 METHODS

6.1 Study parameters/endpoints

6.1.1 Main study parameter/endpoint

Primary outcome measure is the need for any retreatment (also including retreatment that is planned *before* 12 months follow-up, but scheduled *after* 12 months follow-up) (*refer to 5.2* Retreatment in case of treatment failure). Primary outcome will be evaluated at 12 months follow-up.

If a subject refuses one of the study measurements during follow-up, but is otherwise willing to continue study participation, he/she will remain in follow-up for assessment of primary outcome at t=12 months. In case a patient does not adhere to the protocol, or is lost to follow-up, available data from the last included timepoint are used for estimation of the primary endpoint in an intention to treat analysis.

6.1.2 Secondary study parameters/endpoints (if applicable)

Secondary study parameters/outcomes include (see page 26 for follow-up schedule):

- Achalasia symptoms (Eckardt score); Health-related- and disease specific QoL (Pediatric Quality of Life inventory (PedsQL) 4.0, Gastro-intestinal QoL (Pediatric Quality of Life inventory – GI) and Achalasia disease specific QoL questionnaire (DSQoL)) (37-39, 55)

- Reflux symptoms (reflux disease questionnaire, RDQ)
- LA grade (EGD)
- 24 hour pH-impedance (pH-MII) measurement parameters (Table 1)
- HRM parameters (Table 1)
- Procedure times
- Complications (any unwanted events that arise following treatment and/or that are secondary to the treatment)
    - Severe: resulting in admission >24 hours or prolongation of an already planned admission of >24 hours, admission to a medium or intensive care unit, additional endoscopic procedures, blood transfusion or death.
    - Mild: all other complications.

6.2 Randomization, blinding and treatment allocation

When participants are recruited for the study, the time to consider participation is up to four weeks. Before randomization, the patient (if ≥ 12 years) and all legal guardians (if patients <16 years) must have signed the informed consent form.

Randomization will be performed by using a web-based program (Castor electronic data capture (EDC) system) and will be stratified per center, to reach equal number of patients treated with POEM or EBD in each center. To ensure balance of treatment groups, stratified block randomization with randomly selected block sizes in order to minimize selection bias will additionally be performed within each center. Castor EDC will generate the allocation sequence. Allocation concealment is applied to all investigators, clinicians and patients. One blinded central reader will analyze all HRM and pH-MII measurements and a second blinded central reader will analyze all barium esophagrams.

6.3 Study procedures (see figure 2 below for study flow chart, page 26)

Inclusion criteria

The combination of Eckardt symptom score, a timed barium esophagram and HRM gives a complete picture of the esophageal function and are considered standard investigations to diagnose achalasia.

Baseline

At baseline, the following measurements and questionnaires will be performed:

- Standard achalasia care:
    - Body weight and height and body mass index (BMI) including respective *z*-scores
    - Medical history
    - PedsQL 4.0 (41, 42)
- Additional study measurements and questionnaires
    - Disease specific QoL (43)
    - Reflux disease questionnaire (44)
    - PedsQL-GI 3.0 (55)

Questionnaires regarding reflux, achalasia specific quality of life and gastro-intestinal quality of life (see Appendix A) are not currently implemented in standard achalasia care. These two questionnaires take maximum 15 minutes to fill in and are not considered burdensome for patient's psychological well-being.

Follow-up

At 12 months follow-up, the following study procedures are performed in all study participants:

- Barium esophagram
- HRM
- pH-MII
- EGD

In clinical practice, these tests are performed when symptoms or complications suggestive of achalasia recurrence and/or GER. Recent recommendations involve an EGD and pH-MII 1 year post-therapy in order to evaluate the presence of GERD in all achalasia patients. In this study, we also perform a barium esophagram and HRM post-therapy in order to obtain a complete overview of the esophageal function.

Figure 2: Flow chart of study visits and measurements.

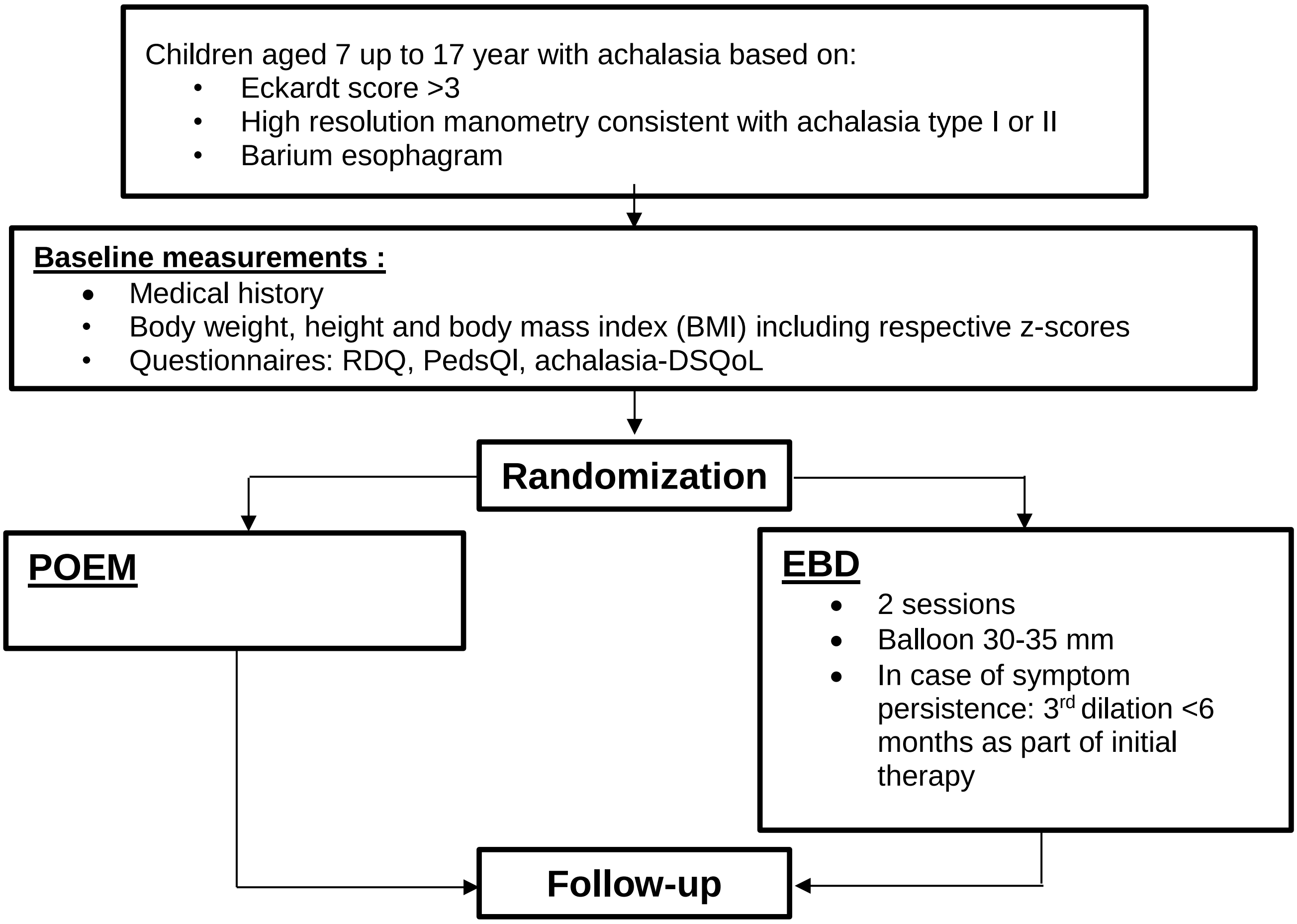

	3 months	6 months	1 year
**Need for relapse therapy**	**Primary endpoint. Patients are asked to report any recurrence of symptoms at all time during study period. At scheduled follow up visits, symptoms will be specifically asked for.**		
**Eckardt Score**	Eckardt score will be monitored on a monthly basis by scheduled phone appointments.		
**Weight, Height & BMI**	X	X	X
**Weight**	Weight will be monitored on a <u>monthly</u> basis in the patient's home-environment with a standardized scale provided by the principal investigator additionally to the in-hospital measurements at the indicated time-points		
**RDQ***	X	X	X
**PedsQL 4.0***	X	X	X
**PedsQL – GI 3.0***	X	X	X
**Achalasia DSQoL***	X	X	X
**Barium esophagram**			X
**HRM***			X

**pH-MII***			X
**EGD+biopsies***			X

Figure 2: flow chart of study visits and measurements. Measurements shown in grey indicate standard achalasia care whereas measurements in green indicate that they are not considered standard care.
*BMI = body mass index; RDQ = reflux disease questionnaire; PedsQoL = Pediatric quality of life inventory; PedsQoL-GI = Pediatric quality of life inventory – Gastro-Intestinal Symptoms; HRM = high resolution manometry; EGD = esophagogastroduodenoscopy

Esophageal Balloon Dilation (EBD)

In this study EBD will be performed by experienced (pediatric) gastroenterologists that have independently performed at least 15 procedures. The procedure will be recorded on video to make evaluation afterwards possible. Pictures to evaluate LA grade of GERD will be taken. Patients will be treated under general anesthesia with endotracheal intubation.

Patients are asked to use a diet with fluids starting at least 48 hours before the procedure, only clear fluids starting 24 hours and nil per mouth starting 8 hours before the procedure.

Initial EBD treatment will consist of a series of two dilatation sessions, scheduled two weeks (10-18 days) apart. In the first session, two dilations of one minute each with a dilatation pressure of respectively 5 and 8 psi will be performed using a single-use low-compliance 30 mm balloon under radiological guidance. The second treatment session is identical but with a larger balloon with a diameter size of 35mm. Patients are discharged without any diet restriction.

If patients in the EBD treatment arm have persisting symptoms , a subsequent dilation with a 35-mm balloon is performed. If a patient has a relapse (recurrent symptoms and evidence of active disease on barium esophagram and/or HRM) a second series of two EBD sessions is performed. This is considered **part of the initial EBD** treatment. If symptom recurrence occurs after a third EBD or second series of two EBDs , this is considered as ‘treatment failure’.

Relapse therapy will then be offered at the discretion of local preferences of the attending physician.

Peroral endoscopic myotomy (POEM)

POEM will be performed by experienced (pediatric) endoscopists, who have independently performed over 15 procedures. Patients are admitted two hours prior to the POEM procedure and are scheduled to be discharged within 48 hours post-procedure. Patients are asked to use a diet with clear fluids starting 48 hours before the procedure and nil per mouth starting 8 hours before the procedure. On the day of the procedure, antibiotics and double dose PPI are administered intravenously (see page 28 and 29 for medicament and dosage). The mouth, throat and esophagus are rinsed with saline and chlorhexidine (40–60 ml). POEM procedures are then performed as described by Inoue et al. The endoscopic procedure will be recorded on video to make evaluation afterwards possible. Pictures to evaluate LA grade of GERD will be taken. A forward-viewing upper endoscope is used with a transparent distal cap. Carbon dioxide gas is used for insufflation during the procedures. An endoscopic dissection knife is used to access the submucosa, to create the submucosal tunnel, and also to divide circular muscle fibers over a minimum length of 6 cm in the esophagus, and 2 cm onto the cardia according to the standards of surgical myotomy. An electrogenerator is used with Endocut Q mode (effect 2) to open the mucosa, and spray coagulation mode (effect 2, 50 watt) to dissect the submucosa and divide the muscle fibers. A coagulating forceps is used for hemostasis as needed. Closure of the mucosal entry site is performed using standard endoscopic clips.

Patients are discharged with a soft diet for 2 weeks.

Reflux surveillance

GERD may develop post-achalasia intervention due to disruption of the LES. A recently performed randomized controlled trial in adult achalasia patients showed a higher incidence of GERD in patients who were treated with POEM (31% grade A; 4% grade B, 8% grade C) compared to those treated with EBD (7% grade A), p=0.002. In a recently published literature review, GERD was present in 8% of children treated with EBD vs 28% of children treated with POEM.(26) However, in these included pediatric studies, GERD was defined with varying criteria and diagnostic tests and many studies were retrospective in design. A true estimate of the occurrence of GERD post-achalasia treatment, will only be possible by prospective evaluation using standardized tools (i.e. EGD with biopsies and pH-MII). As a precautionary measure, all study patients will take PPIs orally in a single daily dose starting at the day after treatment (esomeprazole, <20kg = 10mg/day, ; ≥ 20

kg= 20mg/day for at least one year (+/- 6 weeks) after initial therapy. At 12 months after randomization (+/- 6 weeks), an EGD, HRM and pH-MII will be performed after cessation of PPI 4-6 weeks prior to investigations (Figure 4). In case of abnormal pH-MII and/or EGD results, the attending physician will follow-up and manage GERD according to latest recommendation guidelines pediatric GERD, which may include adjustment of PPI dose and repeat pH-MII/EGD.(46) In case of normal pH-MII and EGD results, PPIs can be ceased.

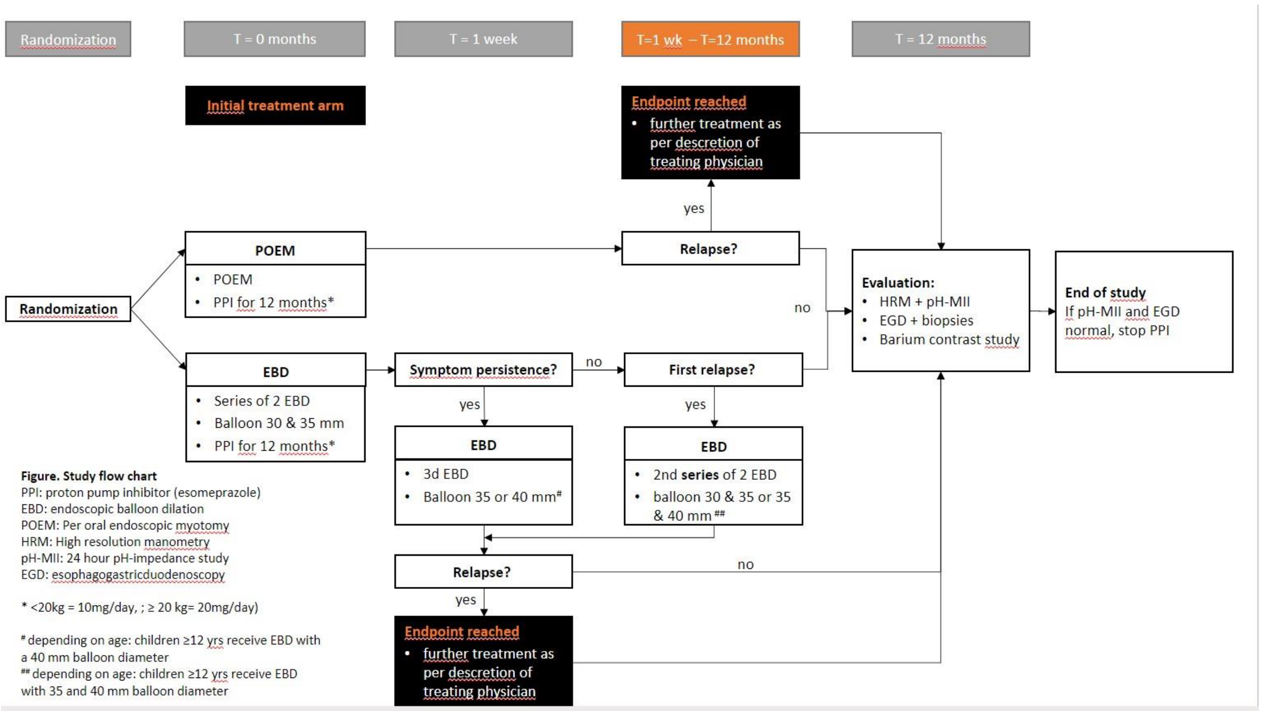

Figure 4: study flow chart

General follow up

Individual procedures during follow up of the study are outlined at page 26 in figure 2.

Apart from these structured follow-up moments, additional tests (e.g. timed barium esophagram,and HRM) can be performed at any time if a subject reports relapse symptoms as per standard achalasia care.

Follow-up visits, measurements and questionnaires should best be performed according to the schedule below. Variations from the schedule will be accepted within limits as follows (table 2):

Table 2: accepted variations from the study schedule:

Study moment	Accepted variations

Second EBD session (part of primary therapy)	1 – 6 weeks after first EBD session
T=3 months follow-up	-2 weeks, + 4 weeks
T=6 months follow-up	+/- 6 weeks
Monthly measurement of weight in the first 12 months	+/- 2 weeks. A maximum of 25% missed measurements is allowed.
HRM, pH-MII and EGD at T=12 months	+/- 6 weeks
Cessation of PPI prior to measurements	- at least 14 days before pH-MII<br>- 4 – 6 weeks before EGD

Questionnaires

The Eckardt score is developed to objectify achalasia symptoms and is the sum of symptom scores for dysphagia, regurgitation, chest pain and weight loss.(47) It is commonly used in both adults as well as children. Each symptom is scored from 0 to 3. The minimum score is 0, the maximum 12. (see Appendix A).

The reflux disease questionnaire is developed to objectify frequency and severity of GER symptoms and is commonly used in pediatric and adult patients.(44)

It consists of 12 items that cover the symptoms heartburn, regurgitation and upper abdominal pain. Each item can be rated from 1-6. A mean score is calculated from all 12 respective answers.

The Pediatric quality of life inventory (PedsQL V4.0) assesses children's and parent's perception of child health related quality of life.(42) The PedsQL consists of four domains (physical, emotional, social and school) and has 23 items. Each item can be rated from 0 – 4. After completion of the questionnaire, the cumulative total score is calculated.

The Gastro-intestinal Pediatric Quality of Life inventory (PedsQL-GI 3.0) assess children's and parents perception of child gastro-intestinal health related quality of life. (55) The PedsQL-GI consist of 74 questions on gastro-intestinal symptoms. Each item can be rated between 0-4, the cumulative total score is calculated.

The achalasia disease specific QoL questionnaire consists of 20 items in three domains (swallow difficulties, family and friends and emotions) that measure disease specific QoL.(43)

Measurements

Timed barium esophagram

In the timed barium esophagram technique, upright frontal spot films of the esophagus are obtained at 1 min after ingestion of low-density (45% weight in volume) barium sulphate. This is a routine clinical test that is used to measure esophageal emptying and esophageal function when (recurrence of) achalasia is suspected.

Barium esophagram protocol:

- Ruler is placed on patient's back in order to measure column height
- Patient swallows barium contrast in upright position
  - 7 – 10 years: 100 cc
  - 10-14 years: 150 cc
  - 14-18 years: 200 cc
- Radiologic films are taken immediately after ingestion of barium (t=0), 1 minutes after barium ingestion
- A barium esophagram is considered abnormal when esophageal stasis occurs ≥1 minute after ingestion.

High resolution manometry (HRM) of the esophagus

HRM of the esophagus (pressure measurement) is the gold standard to diagnose achalasia and is used in clinical practice to evaluate the effect of the treatment. The latest CC is used for the evaluation of esophageal motility disorders and is able to detect three different subtypes of achalasia (type 1 = classic; type 2 = panesophageal pressurization; type 3 = spastic).(39) Intraluminal impedance can be measured in conjunction with pressure (i.e., high-resolution impedance manometry, HRIM). Pressure and impedance channels are then incorporated into one catheter. In some participating centers, only a HRIM catheter is available. As the followed

procedure and patient discomfort (catheter size, duration) for these tests are identical, both HRIM and HRM catheters can be used in this study.

pH-impedance (pH-MII) recording

Adult patients are prone to develop GERD post-EBD or post-myotomy (both laparoscopic and endoscopic) and often have a high esophageal acid exposure time.(7, 27, 48) To objectively evaluate GERD, pH-MII recording is therefore indicated. A pH-MII measurement is a routine clinical test for the assessment of esophageal acid exposure in adults and children. It is currently considered the best available test to correlate symptoms with all types of reflux episodes (i.e. acid and weakly acidic reflux).(49) Multiple studies in adult patients have demonstrated that after myotomy (either LHM or POEM), typical symptoms, such as heartburn and bitter-tasting regurgitation, are poor correlates of objective distal esophageal acid exposure as measured by 24-h pH-MII.(50, 51)

During the 24h pH-MII test, a small catheter is introduced transnasally into the esophagus.(52) If possible, the catheter is placed during EGD under anesthesia. This catheter consists of impedance and pH sensors that measure reflux episodes and esophageal acid exposure. Data are stored on a datalogger that is carried by the patient in a small backpack or shoulderbag. PPI's will be ceased at least 14 days prior to the pH-MII study. See table 1 for pH-MII parameters that are analyzed.

Esophagogastroduodenoscopy (EGD)

EGD is performed under general anesthetics, as per routine clinical care in pediatrics. Biopsies are routinely taken to evaluate the presence of esophagitis.(53) See table 1 for EGD parameters that are analyzed.

After treatment, the main clinical reason to perform EGD is to investigate whether reflux related esophagitis is present. The degree of esophagitis is scored according to the LA classification.(53) PPI therapy is stopped 4-6 weeks before the endoscopy as is standard in clinical practice when the continuing need for PPI therapy is evaluated.

6.4 Withdrawal of individual subjects

Parents and/or patients can redraw subjects from the study at any time for any reason if they wish to do so without any consequences. The investigator can decide to withdraw a subject from the study for urgent medical reasons.

If a subject refuses one of the study measurements during follow-up, but is otherwise willing to continue study participation, he/she will remain in follow-up for assessment of primary outcome at t=12 months. In case a patient does not adhere to the protocol, or is lost to follow-up, available data from the last included timepoint are used for estimation of the primary endpoint in an intention to treat analysis.

6.5 Follow-up of subjects withdrawn from treatment

If a subject gets withdrawn from the study, he/she will have the standard achalasia care, depicted in figure 1. In case of treatment failure after study withdrawal, therapeutic approach will be decided based on shared decision making and the discretion of the attending physician.

If a subject refuses one of the study measurements during follow-up, but is otherwise willing to continue study participation, he/she will remain in follow-up.

6.6 Premature termination of the study

Efficacy and safety data of the study will be monitored independently by the investigators, mainly by the PI, and the DSMB. Safety of the study subjects is the most important issue. After the first 50 patients have reached follow-up moment T=12 months, the DSMB will discuss the data and safety and review the stopping regulations. The study can be terminated early in case of safety issues.

7 SAFETY REPORTING

7.1 Temporary halt for reasons of subject safety

In accordance to section 10, subsection 4, of the WMO, the sponsor will suspend the study if there is sufficient ground that continuation of the study will jeopardize subject health or safety. The sponsor will notify the accredited MREC without undue delay of a temporary halt including the reason for such an action. The study will be suspended pending a further positive decision by the accredited MREC. The investigator will take care that all subjects are kept informed.

7.2 AEs and SAEs

7.2.1 Adverse events (AEs)

Adverse events are defined as any undesirable experience occurring to a subject during the study, whether or not considered related to the intervention. All adverse events reported spontaneously by the subject or observed by the investigator or his staff will be recorded.

7.2.2 Serious adverse events (SAEs)

A serious adverse event is any untoward medical occurrence or effect that

- results in death;
- is life threatening (at the time of the event);
- requires hospitalization or prolongation of existing inpatients' hospitalization of more than one day;
- results in persistent or significant disability or incapacity;
- is a congenital anomaly or birth defect; or
- any other important medical event that did not result in any of the outcomes listed above due to medical or surgical intervention but could have been based upon appropriate judgement by the investigator.

An elective hospital admission will not be considered as a serious adverse event.

The investigator will report all SAEs to the sponsor without undue delay after obtaining knowledge of the events.

7.3 Safety reporting

7.3.1 Adverse events

Adverse events occurring from the first study-related procedure until 30 days after protocol treatment will be recorded in the CRF.

7.3.2 Follow-up of adverse events

All adverse events will be followed until they have abated, or until a stable clinical situation has been reached. Depending on the nature of the event, follow up may require additional tests or medical procedures as indicated, and/or referral to the general physician or a medical specialist.

All AEs must be documented and the outcome must be followed-up until the return to normal or consolidation of the patient's condition.

Subjects withdrawn from the study due to any AE will be followed at least until the outcome is determined even if it implies that the follow-up continues after the patient has left the trial.

7.3.3 Reporting Serious Adverse Events

Serious Adverse Events (SAEs) will be reported from the first study-related procedure until 3 months postpartum.

SAE's must be reported by e-mail to the study project team within 24 hours after the event was reported to the investigator, using the provided SAE report form. This initial report should minimally contain information with respect to the event, associated treatment and patient identification, as described in the detail in the instructions for the SAE report form. If necessary, more detailed information should be provided in a follow-up report within a further 2 business days. SAE's need to be reported until end of study as defined in the protocol.

7.3.4 Follow-up of Serious Adverse Events

All serious adverse events will be followed clinically until they are resolved or until a stable situation has been reached. Depending on the event, follow up may require additional tests or medical procedures as indicated and/or referral to a general physician or a medical specialist.

Follow-up information on SAEs should be reported monthly until recovery or until a stable clinical situation has been reached. The final outcome of the SAE should be reported on a final SAE report.

7.3.5 Processing of Serious Adverse Events

During the course of the study, the sponsor will report in an expedited manner to the Health Authorities, the Ethic Committees in each country in accordance with international and local regulations.

SAE’s that result in death or are life threatening should be reported immediately with a maximal 7 days for a preliminary report with another 8 days for completion of the report. All other SAEs will be reported within a period of maximum 15 days after the sponsor initially received knowledge of the serious adverse events.

If required by national laws or regulations or by the procedures of the authorities, the sponsor will ensure that a six-monthly line listing of all reported SAEs is submitted to the Ethics Committee(s).

7.3.6 Reporting of Safety Issues

The sponsor will promptly notify all concerned investigators, the Ethics Committee(s), and regulatory authorities of findings that could affect adversely patient safety , have impact on the execution of the trial, increase the risk of participation or otherwise alter the EC's approval to continue the trial.

In the occurrence of such an event the sponsor and the investigators will take appropriate urgent safety measures to protect the patients against any immediate hazard. Pending further review, the accredited Ethics Committee will suspend the study, except insofar as suspension would jeopardize patient's health. The local investigator will inform patients participating in the trial.

7.4 Data Safety Monitoring Board (DSMB)

A Data Safety Monitoring Board (DSMB) is established. In total the DSMB will consist of three members, an epidemiologist, a pediatric gastroenterologist and a gastroenterologist. None of the members have a conflict of interest with the sponsor of the study. Personal details of the DSMB members can be found in the DSMB charter which also describes in detail the function, aims and responsibilities of the DSMB. In short the DSMB will act in an independent, expert and advisory capacity to monitor participant safety. The DSMB will evaluate safety of participants after 50 participants have reached 1 year follow up. At this time, the DSMB will be provided with an interim analysis report including outcome measures and all adverse events and serious adverse events that occur during the study.

The advice(s) of the DSMB will only be sent to the sponsor of the study. Should the sponsor decide not to fully implement the advice of the DSMB, the sponsor will send the advice to the reviewing MREC, including a note to substantiate why (part of) the advice of the DSMB will not be followed.

8 STATISTICAL ANALYSIS

All normally distributed data will be presented as means and standard deviations (SD). Data where normal distribution cannot be assumed, will be reported as median and range.

The success rate of the two procedures and their odds ratio will be analysed using logistic regression with adjustment for stratification factors. Superiority of POEM is shown when the primary outcome (need for retreatment) is significantly lower in the POEM treatment arm (defined as $p<0.05$). Data will be shown in point estimates and 95% confidence intervals.

Comparisons between groups will be made using multiple linear regression analysis for continuous data, if necessary after (log)transformation and multiple logistic regression analysis for categorical data for secondary outcomes, adjusting for stratification factors.

9 ETHICAL CONSIDERATIONS

9.1 Regulation statement

The study will be conducted according to the principles of the Declaration of Helsinki (WORLD MEDICAL ASSOCIATION DECLARATION OF HELSINKI Ethical Principles for Medical Research Involving Human Subjects, 2008).

9.2 Recruitment and consent

Patients who visit the Pediatric Gastroenterology departments of the participating centers will be approached. Eligible patients and their parents will be given an explanation of the study. Each patient and his/her legal guardians will receive an information brochure about the study and an informed consent form for participation. They will be given the time needed to read the information and ask questions. Study procedures or randomization are only initiated *after* the informed consent form has been signed. When participants are recruited for the study the time to consider participation in the study is 1 – 4 weeks.

Written informed consent form (ICF) is signed by all legal guardians (parents/caregivers), and/or patient, depending on patient’s age:

- Children < 12 years: ICF signed by all legal guardians.
- Children 12 – 16 years: ICF signed by all legal guardians and patient
- Children ≥ 16 years: ICF signed by patient

In daily practice, the study subject is often joined by only one parent at the outpatient clinic. Therefore, the other parent is allowed to sign the ICF form at home before- or after the hospital visit. As long as both signatures are collected, this is acceptable. In situations where only one parent/legal guardian is known or if one parent is deceased, the absence of the second signature is explained on a Note-to-File or in the patient’s electronic patient file. A copy of the signed informed consent form is given to the participant.

9.3 Objection by minors or incapacitated subjects (if applicable)

Objection of minors will be evaluated according to the ‘Code of conduct relating to expressions of objection by minors participating in medical research’. Approved by the Board of the Netherlands Association for Pediatric Medicine and published in the newsletter no. 3, June 2001. (57)

9.4 Benefits and risks assessment, group relatedness

If a pediatric patient is diagnosed with achalasia, invasive treatment is always needed (i.e. LHM, POEM or EBD). Because EBD and POEM are currently both used as therapeutic options for children with achalasia, children will not have any additional risk when participating in this trial. Although subjects will be managed similarly to patients who are not enrolled in this study, participating this study may be beneficial because of our strict follow-up scheme. Potential treatment failure can therefore be detected in an early stage and additional therapy can be offered early after symptom recurrence. On the other hand, our follow-up scheme can be burdensome as it involves additional time needed to fill in questionnaires that are additional to current standard clinical care.

As only small and often retrospective studies are available about pediatric achalasia therapy, it is unknown how children should best be treated. It is therefore important to compare the two least invasive therapies (i.e. EBD and POEM) in children, to improve future therapeutic strategies in pediatric achalasia.

Risk and complications of EBD

EBD is a frequently performed procedure in children with achalasia but on the long-term, re-dilations are often needed (see introduction and rationale).(19-21, 26) Complications due to EBD are uncommon and include perforation (data from retrospective pediatric studies: n=5/209 and 1/60 respectively(26)), post-procedural pain (n=1/10) and fever (n=2/10). Therapy for

perforations is mostly conservative with food and drink restriction and intravenous antibiotic therapy.

Risk and complications of POEM

POEM is a relatively new procedure in the treatment of achalasia. Results from both pediatric and adult studies indicate that POEM seems to be a promising new treatment for pediatric patients with achalasia, resulting in long-term symptom relief, without serious adverse events and with potential beneficial influence on QoL.

POEM has been shown a feasible and safe procedure in children.(10, 11, 16, 36-38) The largest pediatric study included 27 individuals with success achieved in 26 during two years of follow-up in the absence of serious adverse events or special technical difficulties.(10)

Serious adverse events that could theoretically occur during the procedure are bleeding or esophageal perforation. Treatment of these complications can instantaneously be performed during the procedure by clipping the bleeding vessel or perforation. In exceptional cases a surgical procedure could be needed. The largest cohort study in children (n=44, follow-up 1 – 53 months) reported only mild intraoperative complications including retroperitoneal CO2 (7%), capnoperitoneum (3%), and mucosal injury (1%). Pneumoperitoneum can be relieved by a puncture during the procedure. Postoperative retroperitoneal CO2 is usually self-limiting and additional treatment is not needed. So far the only complications that occurred in the treated (adult and pediatric) patients of the Amsterdam UMC, location Academic Medical Center (AMC) consisted of minor bleedings that were stopped during the procedure and one patient developed a pneumoperitoneum which was relieved during the procedure.

All the additional measurements that are performed before and after treatment are safe procedures and routinely performed in the clinical setting. Possible complications are mainly due to placement of the catheters and endoscope. The catheters can give minor discomfort in the nose and pharynx. Furthermore in rare cases a mucosal bleeding of the nose, caused by the catheter, can occur which never needs additional treatment. The endoscope can cause some discomfort in the

pharynx after the procedure.

Risks due to COVID-19 pandemic

Participants of this study will not be exposed to additional Covid-19 risks compared to peers with achalasia who do not participate in this study, as the studied procedures (EBD and POEM) are part of standard medical care. If necessary, outpatient clinic visits at T=3, 6 and 12 months can be rescheduled as a “video consult”. If, due to COVID-19, additional testing for study procedures at T=12 months are not possible to perform, we will note this in our database as a protocol deviation. If a participant experiences recurrent symptoms and additional testing is needed as part of standard clinical care, these measurements will be scheduled and additional precautionary measures for Covid-19 will be taken.

9.5 Compensation for injury

The sponsor/investigator has a liability insurance which is in accordance with article 7, subsection 6 of the WMO.

The sponsor (also) has an insurance which is in accordance with the legal requirements in the Netherlands (Article 7 WMO and the Measure regarding Compulsory Insurance for Clinical Research in Humans of 23th June 2003). This insurance provides cover for damage to research subjects through injury or death caused by the study.

The insurance applies to the damage that becomes apparent during the study or within 4 years after the end of the study.

9.6 Incentives

Patients participating in this study will not receive any form of financial compensation

10 ADMINISTRATIVE ASPECTS, MONITORING AND PUBLICATION

10.1 Handling and storage of data and documents

The data of the subjects are coded in order of participation. The code can only be seen by the investigators. The code and the data are stored in different locations. Data is captured in an online medical research database (Castor) that is ISO 27001 and ISO 9001 certified.
Qualified authorities can get insight in code and data, but only when accompanied by the investigators. Data will be stored 20 years after closure of the trial, in order to enable long-term follow-up of this cohort until all patients have reached adulthood. All personal data is protected conform the reules of the General Data Protection Regulation.

10.2 Handling and storage of data and documents

Monitoring in the Amsterdam UMC will be performed by the Clinical Research Unit of the Amsterdam UMC-AMC**.**. Monitoring of other sites will occur locally according to local legislation and agreements with the local ethical committee.
Data monitoring will be performed by a certified clinical research associate of the participating institute or its delegate. The monitor will compare the data entered into the database with the hospital or clinic records (source documents). The nature and location of all source documents will be identified to ensure that all sources of original data required to complete the database are known to the investigational staff and are accessible for verification. Frequency of monitoring will be further discussed with the monitoring party.

10.3 Amendments

Amendments are changes made to the research after a favorable opinion by the accredited MREC has been given. All amendments will be notified to the MREC that gave a favorable opinion.

10.4 Annual progress report

The sponsor/investigator will submit a summary of the progress of the trial to the accredited MREC once a year. Information will be provided on the date of inclusion of the first subject,

numbers of subjects included and numbers of subjects that have completed the trial, serious adverse events/ serious adverse reactions, other problems, and amendments.

10.5 Temporary halt and (prematurely) end of study report

The investigator/sponsor will notify the accredited MREC of the end of the study within a period of 8 weeks. The end of the study is defined as the patient's last visit.

The sponsor will notify the MREC immediately of a temporary halt of the study, including the reason of such an action.

In case the study is ended prematurely, the sponsor will notify the accredited MREC within 15 days, including the reasons for the premature termination.

Within one year after the end of the study, the investigator/sponsor will submit a final study report with the results of the study, including any publications/abstracts of the study, to the accredited MREC.

10.6 Public disclosure and publication policy

The General Assessment and Registration Form (ABR-form) will be filled in to provide transparency with regard to this trial. This study is also registered in the ISRCTN (www.isrctn.com). The study results will be made public by submission/publication in international journals and at scientific meetings.

11 REFERENCES

1. Nurko S, Rosen R. Other motor disorders. In: Walker WA, editor. Pediatric gastrointestinal disease. Hamilton: BC Decker; 2004. p. 424-62.
2. Smits M, van Lennep M, Vrijlandt R, Benninga M, Oors J, Houwen R, et al. Pediatric Achalasia in the Netherlands: Incidence, Clinical Course, and Quality of Life. The Journal of pediatrics. 2016;169:110-5.e3.
3. Marlais M, Fishman JR, Fell JM, Haddad MJ, Rawat DJ. UK incidence of achalasia: an 11-year national epidemiological study. Archives of disease in childhood. 2011;96(2):192-4.
4. Hung Y-C, Westfal ML, Chang DC, Kelleher CM. Heller myotomy is the optimal index procedure for esophageal achalasia in adolescents and young adults. Surgical Endoscopy. 2018.
5. Inoue H, Minami H, Kobayashi Y, Sato Y, Kaga M, Suzuki M, et al. Peroral endoscopic myotomy (POEM) for esophageal achalasia. Endoscopy. 2010;42(4):265-71.
6. Zhang X, Modayil RJ, Friedel D, Gurram KC, Brathwaite CE, Taylor SI, et al. Per-oral endoscopic myotomy in patients with or without prior Heller's myotomy: comparing long-term outcomes in a large U.S. single-center cohort (with videos). Gastrointest Endosc. 2018;87(4):972-85.
7. Akintoye E, Kumar N, Obaitan I, Alayo QA, Thompson CC. Peroral endoscopic myotomy: a meta-analysis. Endoscopy. 2016;48(12):1059-68.
8. Evensen H, Kristensen V, Larssen L, Sandstad O, Hauge T, Medhus AW. Outcome of peroral endoscopic myotomy (POEM) in treatment-naive patients. A systematic review. Scandinavian Journal of Gastroenterology. 2019:1-7.
9. Ponds FA, Fockens P, Lei A, Neuhaus H, Beyna T, Kandler J, et al. Effect of Peroral Endoscopic Myotomy vs Pneumatic Dilation on Symptom Severity and Treatment Outcomes Among Treatment-Naive Patients With Achalasia: A Randomized Clinical TrialPeroral Endoscopic Myotomy vs Pneumatic Dilation for Treatment-Naive Patients With AchalasiaPeroral Endoscopic Myotomy vs Pneumatic Dilation for Treatment-Naive Patients With Achalasia. JAMA. 2019;322(2):134-44.
10. Chen WF, Li QL, Zhou PH, Yao LQ, Xu MD, Zhang YQ, et al. Long-term outcomes of peroral endoscopic myotomy for achalasia in pediatric patients: a prospective, single-center study. Gastrointestinal endoscopy. 2015;81(1):91-100.
11. Nabi Z, Ramchandani M, Reddy DN, Darisetty S, Kotla R, Kalapala R, et al. Per Oral Endoscopic Myotomy in Children with Achalasia Cardia. Journal of neurogastroenterology and motility. 2016;22(4):613-9.
12. Miao S, Wu J, Lu J, Wang Y, Tang Z, Zhou Y, et al. Peroral Endoscopic Myotomy in Children With Achalasia: A Relatively Long-term Single-center Study. Journal of pediatric gastroenterology and nutrition. 2018;66(2):257-62.
13. Kethman WC, Thorson CM, Sinclair TJ, Berquist WE, Chao SD, Wall JK. Initial experience with peroral endoscopic myotomy for treatment of achalasia in children. Journal of pediatric surgery. 2017.
14. Nabi Z, Ramchandani M, Chavan R, Darisetty S, Kalapala R, Shava U, et al. Outcome of peroral endoscopic myotomy in children with achalasia. 2019.
15. Lee Y, Brar K, Doumouras AG, Hong D. Peroral endoscopic myotomy (POEM) for the treatment of pediatric achalasia: a systematic review and meta-analysis. Surgical Endoscopy. 2019;33(6):1710-20.
16. Tan Y, Zhu H, Li C, Chu Y, Huo J, Liu D. Comparison of peroral endoscopic myotomy and endoscopic balloon dilation for primary treatment of pediatric achalasia. Journal of pediatric surgery. 2016;51(10):1613-8.
17. Kahrilas PJ, Bredenoord AJ, Fox M, Gyawali CP, Roman S, Smout AJ, et al. The Chicago Classification of esophageal motility disorders, v3.0. Neurogastroenterol Motil. 2015;27(2):160-74.
18. van Lennep M, van Wijk MP, Omari TIM, Salvatore S, Benninga MA, Singendonk MMJ, et al. Clinical Management of Pediatric Achalasia: A Survey of Current Practice. Journal of pediatric gastroenterology and nutrition. 9000;Publish Ahead of Print.
19. Boyle JT, Cohen S, Watkins JB. Successful treatment of achalasia in childhood by pneumatic dilatation. The Journal of pediatrics. 1981;99(1):35-40.

20. Di Nardo G, Rossi P, Oliva S, Aloi M, Cozzi DA, Frediani S, et al. Pneumatic balloon dilation in pediatric achalasia: efficacy and factors predicting outcome at a single tertiary pediatric gastroenterology center. Gastrointestinal endoscopy. 2012;76(5):927-32.
21. Perisic VN, Scepanovic D, Radlovic N. Nonoperative treatment of achalasia. Journal of pediatric gastroenterology and nutrition. 1996;22(1):45-7.
22. Khan AA, Shah SWH, Alam A, Butt AK, Shafqat F. Efficacy of rigiflex balloon dilatation in 12 children with achalasia: a 6-month prospective study showing weight gain and symptomatic improvement. Diseases of the Esophagus. 2002;15(2):167-70.
23. Pastor AC, Mills J, Marcon MA, Himidan S, Kim PC. A single center 26-year experience with treatment of esophageal achalasia: is there an optimal method? Journal of pediatric surgery. 2009;44(7):1349-54.
24. Saliakellis E, Thapar N, Roebuck D, Cristofori F, Cross K, Kiely E, et al. Long-term outcomes of Heller's myotomy and balloon dilatation in childhood achalasia. European journal of pediatrics. 2017;176(7):899-907.
25. Zagory JA, Golden JM, Demeter NE, Nguyen Y, Ford HR, Nguyen NX. Heller Myotomy Is Superior to Balloon Dilatation or Botulinum Injection in Children with Achalasia: A Two-Center Review. Journal of laparoendoscopic & advanced surgical techniques Part A. 2016;26(6):483-7.
26. van Lennep M, van Wijk MP, Omari TIM, Benninga MA, Singendonk MMJ. Clinical management of pediatric achalasia. Expert Rev Gastroenterol Hepatol. 2018;12(4):391-404.
27. Moonen A, Annese V, Belmans A, Bredenoord AJ, Bruley des Varannes S, Costantini M, et al. Long-term results of the European achalasia trial: a multicentre randomised controlled trial comparing pneumatic dilation versus laparoscopic Heller myotomy. Gut. 2016;65(5):732-9.
28. Evensen H, Kristensen V, Larssen L, Sandstad O, Hauge T, Medhus AW. Outcome of peroral endoscopic myotomy (POEM) in treatment-naive patients. A systematic review. Scandinavian journal of gastroenterology. 2019;54(1):1-7.
29. Marlais M, Fishman JR, Fell JM, Rawat DJ, Haddad MJ. Health-related quality of life in children with achalasia. J Paediatr Child Health. 2011;47(1-2):18-21.
30. Enestvedt BK, Williams JL, Sonnenberg A. Epidemiology and practice patterns of achalasia in a large multi-centre database. Alimentary pharmacology & therapeutics. 2011;33(11):1209-14.
31. Zendehdel K, Nyren O, Edberg A, Ye W. Risk of esophageal adenocarcinoma in achalasia patients, a retrospective cohort study in Sweden. The American journal of gastroenterology. 2011;106(1):57-61.
32. Pandolfino JE, Kwiatek MA, Nealis T, Bulsiewicz W, Post J, Kahrilas PJ. Achalasia: a new clinically relevant classification by high-resolution manometry. Gastroenterology. 2008;135(5):1526-33.
33. Meyer A, Catto-Smith A, Crameri J, Simpson D, Alex G, Hardikar W, et al. Achalasia: Outcome in children. Journal of gastroenterology and hepatology. 2017;32(2):395-400.
34. Campos GM, Vittinghoff E, Rabl C, Takata M, Gadenstatter M, Lin F, et al. Endoscopic and surgical treatments for achalasia: a systematic review and meta-analysis. Ann Surg. 2009;249(1):45-57.
35. Pacilli M, Davenport M. Results of Laparoscopic Heller's Myotomy for Achalasia in Children: A Systematic Review of the Literature. Journal of laparoendoscopic & advanced surgical techniques Part A. 2017;27(1):82-90.
36. Li C, Tan Y, Wang X, Liu D. Peroral endoscopic myotomy for treatment of achalasia in children and adolescents. Journal of pediatric surgery. 2015;50(1):201-5.
37. Li QL, Chen WF, Zhou PH, Yao LQ, Xu MD, Hu JW, et al. Peroral endoscopic myotomy for the treatment of achalasia: a clinical comparative study of endoscopic full-thickness and circular muscle myotomy. J Am Coll Surg. 2013;217(3):442-51.
38. Tang X, Gong W, Deng Z, Zhou J, Ren Y, Zhang Q, et al. Usefulness of peroral endoscopic myotomy for treating achalasia in children: experience from a single center. Pediatric surgery international. 2015;31(7):633-8.
39. Kahrilas PJ, Bredenoord AJ, Fox M, Gyawali CP, Roman S, Smout AJ, et al. The Chicago Classification of esophageal motility disorders, v3.0. Neurogastroenterology and motility : the official journal of the European Gastrointestinal Motility Society. 2015;27(2):160-74.

40. Singendonk MMJ, Kritas S, Cock C, Ferris L, McCall L, Rommel N, et al. Applying the Chicago Classification criteria of esophageal motility to a pediatric cohort: effects of patient age and size. 2014;26(9):1333-41.
41. Varni JW, Burwinkle TM, Seid M, Skarr D. The PedsQL 4.0 as a pediatric population health measure: feasibility, reliability, and validity. Ambul Pediatr. 2003;3(6):329-41.
42. Varni JW, Seid M, Kurtin PS. PedsQL 4.0: reliability and validity of the Pediatric Quality of Life Inventory version 4.0 generic core scales in healthy and patient populations. Med Care. 2001;39(8):800-12.
43. Marlais M, Fishman JR, Rawat DJ, Haddad M. Development and validation of a disease-specific quality-of-life measure for children with achalasia. Eur J Pediatr Surg. 2010;20(2):92-4.
44. Rey E, Barceló M, Zapardiel J, Sobreviela E, Muñoz M, Díaz-Rubio M. Is the reflux disease questionnaire useful for identifying GERD according to the Montreal definition? BMC Gastroenterology. 2014;14:17-.
45. Inoue H, Sato H, Ikeda H, Onimaru M, Sato C, Minami H, et al. Per-Oral Endoscopic Myotomy: A Series of 500 Patients. Journal of the American College of Surgeons. 2015;221(2):256-64.
46. Rosen R, Vandenplas Y, Singendonk M, Cabana M, DiLorenzo C, Gottrand F, et al. Pediatric Gastroesophageal Reflux Clinical Practice Guidelines: Joint Recommendations of the North American Society for Pediatric Gastroenterology, Hepatology, and Nutrition and the European Society for Pediatric Gastroenterology, Hepatology, and Nutrition. 2018;66(3):516-54.
47. Eckardt VF. Clinical presentations and complications of achalasia. Gastrointest Endosc Clin N Am. 2001;11(2):281-92, vi.
48. Leeuwenburgh I, Van Dekken H, Scholten P, Hansen BE, Haringsma J, Siersema PD, et al. Oesophagitis is common in patients with achalasia after pneumatic dilatation. Alimentary pharmacology & therapeutics. 2006;23(8):1197-203.
49. Singendonk MM, Benninga MA, van Wijk MP. Reflux monitoring in children. Neurogastroenterol Motil. 2016;28(10):1452-9.
50. Familiari P, Gigante G, Marchese M, Boskoski I, Tringali A, Perri V, et al. Peroral Endoscopic Myotomy for Esophageal Achalasia: Outcomes of the First 100 Patients With Short-term Follow-up. Annals of surgery. 2016;263(1):82-7.
51. Gholoum S, Feldman LS, Andrew CG, Bergman S, Demyttenaere S, Mayrand S, et al. Relationship between subjective and objective outcome measures after Heller myotomy and Dor fundoplication for achalasia. Surg Endosc. 2006;20(2):214-9.
52. Bredenoord AJ. Impedance-pH monitoring: new standard for measuring gastro-oesophageal reflux. Neurogastroenterol Motil. 2008;20(5):434-9.
53. Lundell LR, Dent J, Bennett JR, Blum AL, Armstrong D, Galmiche JP, et al. Endoscopic assessment of oesophagitis: clinical and functional correlates and further validation of the Los Angeles classification. Gut. 1999;45(2):172-80.
54. Boeckxstaens GE, Annese V, des Varannes SB, Chaussade S, Costantini M, Cuttitta A, et al. Pneumatic dilation versus laparoscopic Heller's myotomy for idiopathic achalasia. N Engl J Med. 2011;364(19):1807-16.
55. Varni JW, Bendo CB, Denham J, Shulman RJ, Self MM, Neigut DA, Nurko S, Patel AS, Franciosi JP, Saps M, Verga B, Smith A, Yeckes A, Heinz N, Langseder A, Saeed S, Zacur GM, Pohl JF. PedsQL gastrointestinal symptoms module: feasibility, reliability, and validity. J Pediatr Gastroenterol Nutr. 2014 Sep;59(3):347-55. doi: 10.1097/MPG.0000000000000414. PMID: 24806837.
56. Hansson L, Hedner T, Dahlöf B. Prospective randomized open blinded end-point (PROBE) study. A novel design for intervention trials. Prospective Randomized Open Blinded End-Point. Blood Press. 1992;1(2):113-9.
57. Code of conduct relating to expressions of objection by minors participating in medical research.Code+of+conduct+relating+to+expressions+of+objection+by+minors+participating+in+medical+research.pdf
