# Supplementary material for: Protocol for an international multicenter randomized controlled trial assessing treatment success and safety of peroral endoscopic myotomy vs endoscopic balloon dilation for the treatment of achalasia in children
Source: PLoS One. 2023 Oct 5;18(10):e0286880. doi: 10.1371/journal.pone.0286880 (PMC10553306; doi:10.1371/journal.pone.0286880)
Supplement: S2 File — (PDF) [file pone.0286880.s003.pdf]

Amsterdam UMC
Universitair Medische Centra

Aan de heer prof.dr. M.A. Benninga
Kindergeneeskunde
H7-248

Medisch Ethische Toetsingscommissie AMC
TK0-270

Amsterdam, 21 juni 2021
ons kenmerk: 2021_028#B2021374
betreft: **Positief besluit**
NL68967.018.20
Peroral endoscopie myotomy (POEM) vs endoscopic balloon dilation (EBD) for the treatment of achalasia in children

Geachte heer Benninga,

De METC AMC heeft zich, op grond van artikel 2, lid 2, sub a van de Wet medisch-wetenschappelijk onderzoek met mensen (WMO) beraden over bovengenoemd onderzoeksdossier.
Wij delen u gaarne mee dat onze commissie
- tot oordelen bevoegd krachtens artikel 2, tweede lid, onder a, van de Wet medisch-wetenschappelijk onderzoek met mensen (WMO);
- werkzaam volgens de ICH-GCP richtlijnen;
- op grond van de haar voorgelegde stukken als hierna vermeld;
- gelet op artikel 3 en artikel 4 van de WMO;
- gelet op artikel 5 en 6;
- vastgesteld hebbende dat voorzien is in de dekking van een aansprakelijkheidsverzekering als bedoeld in artikel 7, lid 9 van de WMO,

heeft besloten tot een positief oordeel over dit protocol en de uitvoering daarvan in het Amsterdam UMC, locatie AMC en locatie VUmc.

Voorts hebben wij vastgesteld dat voor het onderzoek een verzekering is afgesloten conform de WMO door AMC ten behoeve van proefpersonen van dit onderzoek.

In de beoordeling betrokken documenten:
A1 aanbiedingsemail d.d. 25 januari 2021
A1 aanbiedingsbrief d.d. 25 januari 2021
A1 aanbiedingsemail d.d. 14 april 2021
A1 aanbiedingsemail d.d. 19 april 2021
A1 aanbiedingsemail d.d. 19 mei 2021
A1 aanbiedingsemail d.d. 15 juni 2021
A1 aanbiedingsemail d.d. 17 juni 2021
B1 ABR-formulier NL68967.018.20 versie 08 d.d. 16 juni 2021
B2 AMC appendix, getekend d.d. 14 april 2021 TC
C1 protocol versie 2 d.d. 14 april 2021 TC
E1 E2 proefpersoneninformatie en toestemmingsverklaring ouders_verzorgers versie 2 d.d. 14 april 2021 TC
E1 E2 proefpersoneninformatie en toestemmingsverklaring kinderen 12-15 jaar versie 2 d.d. 14 april 2021 TC
E1 E2 proefpersoneninformatie en toestemmingsverklaring kinderen 16-18 jaar versie 2 d.d. 14 april 2021 TC
E1 proefpersoneninformatie kinderen tot 12 jaar versie 2 d.d. 14 april 2021 TC
F1 vragenlijst achalasie kwaliteit van leven kind 7-12 jaar versie, ongedateerd
F1 vragenlijst reflux disease questionnaire (RDQ) versie, 2000
F1 vragenlijst PedsQL-4.0-Core-Adol kind 8-12 jaar NL versie 4.0 d.d. maart 2004
F1 vragenlijst PedsQL-4.0-Core-Adol kind 13-18 jaar NL versie 4.0 d.d. maart 2004
F1 vragenlijst Eckardt score versie, 2001
F1 vragenlijst achalasie kwalitieit van leven kind 12-18 jaar versie, ongedateerd

AMC
Meibergdreef 9
1105 AZ Amsterdam
Postbus 22660
1100 DD Amsterdam
T +31(0)20 566 9111
www.amc.nl

H1 CV onafh. arts Koot, AMC
H2 CV coord. onderzoeker van Wijk, AMC
I1 lijst deelnemende centra met hoofdonderzoekers versie 1 d.d. 7 april 2020
I2 onderzoeksverklaring VUmc d.d. 19 mei 2021
I3 CV hoofdonderzoeker van Wijk, VUmc
I3 CV hoofdonderzoeker Benninga, AMC
K3 onderzoekscontract draft versie 1 d.d. 29 mei 2020
K5 DSMB d.d. 22 januari 2021
K6 bevestiging centrale CRU monitoring d.d. 16 april 2020
K6 risk assessment d.d. 1 april 2020
K6 stralingsadvies d.d. 13 september 2019

Het onderzoeksdossier, aan ons ter beoordeling voorgelegd op 25 januari 2021 is besproken in de vergadering van onze commissie van 18 februari 2021 en vervolgens aan de orde geweest in de vergadering van het dagelijks bestuur van onze commissie van 28 april 2021, na ontvangst van aangepaste documenten op 14 april 2021. De commissie gaat akkoord met de deelname van kinderen omdat de techniek zich bewezen heeft bij volwassenen. Er werden vragen gesteld over onder andere de belasting en methodologie en de proefpersoneninformatie. Deze werden voldoende beantwoord en de afhandeling werd gemandateerd aan de secretaris. Deze heeft de stukken voorgelegd op 19 mei 2021 en 17 juni 2021 gezien en vastgesteld dat tegemoet gekomen is aan de opmerkingen van de commissie.

U dient onze commissie op de hoogte te stellen van de daadwerkelijke start van het onderzoek, van de (al dan niet voortijdige) beëindiging daarvan, en van tijdens het onderzoek optredende onverwachte complicaties. Voorts dienen eventuele protocolwijzigingen ter beoordeling aan onze commissie te worden voorgelegd. U dient tevens ons jaarlijks een voortgangsrapportage betreffende het onderzoek te doen toekomen, voor het eerst binnen een jaar na dagtekening van dit besluit.

Wij wijzen u erop dat op grond van artikel 23 van de Wet medisch-wetenschappelijk onderzoek met mensen degene wiens belang rechtstreeks bij een besluit van de METC is betrokken, daartegen binnen zes weken na de dag waarop het besluit bekend is gemaakt, een administratief beroepschrift kan indienen bij de Centrale Commissie Mensgebonden Onderzoek. Een dergelijk administratief beroepschrift dient geadresseerd te worden aan: CCMO, Postbus 16302, 2500 BH Den Haag.

Ten slotte brengen wij onder uw aandacht dat dit besluit zijn geldigheid verliest als het onderzoek niet binnen twee jaar na dagtekening van deze brief is gestart.

Ten tijde van de beoordeling van dit project was de commissie als volgt samengesteld:

prof.dr. J.A. Swinkels	:	voorzitter, psychiater
mw.drs. G.H.M. van Ammers	:	lid dat onderzoek beoordeelt vanuit de invalshoek van de proefpersoon
mw. dr. I.H. Bartelink	:	plv. lid ziekenhuisapotheker, klinisch farmacoloog
dr. H. van den Berg	:	lid kinderarts
dr. P.M. Bet	:	plv. lid ziekenhuisapotheker, klinisch farmacoloog
mw.prof.dr. M.A. Boermeester	:	chirurg
prof. dr. A.J. Bredenoord	:	maag darm lever arts
prof.dr. M.G.W. Dijkgraaf	:	plv. lid, methodoloog
mw. J.M.M. Dijkstra	:	lid dat onderzoek beoordeelt vanuit de invalshoek van de proefpersoon
prof.mr.dr. J.K.M. Gevers	:	plv. jurist
prof.dr J.W. Groothoff	:	plv. kinderarts
dr.ir. J.M. den Harder	:	klinisch fysicus
mw.prof.dr. M.D. Hazenberg	:	internist-hematoloog
dr. J.Ph. de Jong	:	plv. lid, ethicus
dr. R.E. Jonkers	:	longarts/plv. lid klinisch farmacoloog
dr. M.J.W. Koelemay	:	vaatchirurg
mw.mr. E.J. Kranendonk	:	jurist
prof.dr. R.A.A. Mathôt	:	ziekenhuisapotheker, klinisch farmacoloog
dr. J.T.M. van der Meer	:	internist-infectioloog
dr. P.J Nederkoorn	:	neuroloog
mw.mr. W. Paping-Kool	:	plv. jurist

AMC en VUmc werken samen in Amsterdam UMC
AMC
Meibergdreef 9
1105 AZ Amsterdam
Postbus 22660
1100 DD Amsterdam
T +31(0)20 566 9111
www.amc.nl

prof.dr. A.J.P.M. Smout	:	maag darm lever arts
mw.mr. L.M. Spittuler	:	lid dat onderzoek beoordeelt vanuit de invalshoek van de proefpersoon
prof.dr. J. Stam	:	neuroloog
dr. H.L. Tan	:	cardioloog
prof.dr. J.G.P. Tijssen	:	plv. lid, klinisch epidemioloog
prof.dr. D.L. Willems	:	plv. medisch ethicus
prof.dr. A.H. Zwinderman	:	biostatisticus.

Met vriendelijke groet,
namens de Medisch Ethische Toetsingscommissie AMC,

Mw. dr. C.L. van der Wilt
ambtelijk secretaris

Zo lang de beperkende maatregelen als gevolg van het coronavirus gelden zullen de besluiten van de METC niet worden voorzien van een natte handtekening. De besluiten worden digitaal verstuurd. Indien u na het intrekken van de maatregelen alsnog een ondertekend besluit nodig heeft, verneemt de METC dit graag.

c.c. CCMO (pdf via TOL)
c.c. pdf per e-mail M.van Lennep, I.J.Zuidgeest
c.c. apotheek (pdf per e-mail)
c.c. EPIC (registratie AMC)
c.c. EPIC (registratie VUmc)

AMC en VUmc werken samen in Amsterdam UMC

AMC
Meibergdreef 9
1105 AZ Amsterdam

Postbus 22660
1100 DD Amsterdam

T +31(0)20 566 9111
www.amc.nl
