# Supplementary material for: Protocol for an international multicenter randomized controlled trial assessing treatment success and safety of peroral endoscopic myotomy vs endoscopic balloon dilation for the treatment of achalasia in children
Source: PLoS One. 2023 Oct 5;18(10):e0286880. doi: 10.1371/journal.pone.0286880 (PMC10553306; doi:10.1371/journal.pone.0286880)
Supplement: Attachment — Submitted filename: Response to Reviewers.docx [file pone.0286880.s004.docx]

Dear Editor,

Hereby we would like to respond to all points. The manuscript is amended to meet all journal requirements. After review, no changes were made to the references. All minor revisions were implemented in the updated manuscript. Details and further explanation can be found below.

Thank you in advance for considering publication.

Kind regards,

Michiel van Wijk

Carlijn Mussies & Marinde van Lennep

Journal requirements:

When submitting your revision, we need you to address these additional requirements.

1. Please ensure that your manuscript meets PLOS ONE's style requirements, including those for file naming. The PLOS ONE style templates can be found at

<https://journals.plos.org/plosone/s/file?id=wjVg/PLOSOne_formatting_sample_main_body.pdf> and [https://journals.plos.org/plosone/s/file?id=ba62/PLOSOne_formatting_sample_title_authors_affiliations.pdf](https://eur04.safelinks.protection.outlook.com/?url=https%3A%2F%2Fjournals.plos.org%2Fplosone%2Fs%2Ffile%3Fid%3Dba62%2FPLOSOne_formatting_sample_title_authors_affiliations.pdf&data=05%7C01%7Cc.mussies%40amsterdamumc.nl%7Cfb39bcfadddd4cf0c24b08db1e41d68c%7C68dfab1a11bb4cc6beb528d756984fb6%7C0%7C0%7C638137040345739309%7CUnknown%7CTWFpbGZsb3d8eyJWIjoiMC4wLjAwMDAiLCJQIjoiV2luMzIiLCJBTiI6Ik1haWwiLCJXVCI6Mn0%3D%7C3000%7C%7C%7C&sdata=%2Fam9WNJMTJdUeOpyrXG3scfLBcitNkFmEHUR3%2Bmjjxo%3D&reserved=0).

**The manuscript was amended to meet all style requirements**.

2. Thank you for submitting your clinical trial to PLOS ONE and for providing the name of the registry and the registration number. The information in the registry entry suggests that your trial was registered after patient recruitment began. PLOS ONE strongly encourages authors to register all trials before recruiting the first participant in a study. As per the journal’s editorial policy, please include in the Methods section of your paper: 1) your reasons for your delay in registering this study (after enrolment of participants started); 2) confirmation that all related trials are registered by stating: “The authors confirm that all ongoing and related trials for this drug/intervention are registered.

**The trial was registered after the first inclusion because the process of registration took longer than expected. Only one patient was included before registration was completed. We can confirm that all ongoing and related trials are registered. This is also stated in the manuscript.**

3. Thank you for stating the following financial disclosure:

“For Wishdom Foundation

<https://www.forwishdom.org/>”

Please state what role the funders took in the study.  If the funders had no role, please state: ""The funders had no role in study design, data collection and analysis, decision to publish, or preparation of the manuscript.""

If this statement is not correct you must amend it as needed.

Please include this amended Role of Funder statement in your cover letter; we will change the online submission form on your behalf.

4. Thank you for stating the following in the Acknowledgments/ Funding Section of your manuscript:

“This study is funded by for Wishdom.”

We note that you have provided funding information that is not currently declared in your Funding Statement. However, funding information should not appear in the Acknowledgments section or other areas of your manuscript. We will only publish funding information present in the Funding Statement section of the online submission form.

Please remove any funding-related text from the manuscript and let us know how you would like to update your Funding Statement. Currently, your Funding Statement reads as follows:

“For Wishdom Foundation

<https://www.forwishdom.org/>”

Please include your amended statements within your cover letter; we will change the online submission form on your behalf.

**The updated financial statement can be found in the cover letter. We can confirm that the funders had no role in study design, data collection and analysis, decision to publish, or preparation of the manuscript.**

5. Your ethics statement should only appear in the Methods section of your manuscript. If your ethics statement is written in any section besides the Methods, please delete it from any other section.

**The ethical considerations are removed from any other section besides the method section.**

6. Please include a separate caption for each figure in your manuscript.

7. Please include a copy of Table 1 which you refer to in your text on page 11.

**Both are added to the manuscript.**

Minor revisions suggested by reviewer #1:

1- Line 173: Indicate the statistical testing method which achieves 80% power.

**A two group χ² test with a 5% two-sided significance level will have 80% power to detect the difference between a Group 1 proportion, π₁, of 0.725 and a Group 2 proportion, π₂ of 0.925 (odds ratio of 4.678) when the sample size in each group is 56. (nQuery (version 9)). This is added to the manuscript in the corresponding line.**

2- Lines 341-6: Provide corresponding percentages for the fractions shown.

**All corresponding percentages are added to the manuscript.**

3- Line 386 Statistical analysis: Indicate how categorical variables will be summarized. Typically frequencies and corresponding percentages are provided.

**Categorical data will be stated using frequencies and corresponding percentages. This is added to the manuscript in the paragraph “statistical analysis”.**

4- State how adverse event data will be summarized. Indicate if a standard method for collecting adverse events will be implemented such as the Common Terminology Criteria for Adverse Events (CTCAE) version 5.

**Adverse events will be defined as any undesirable experience occurring to a subject during the study, whether or not considered related to the intervention. All adverse events reported spontaneously by the subject or observed by the investiga­tor or his staff will be recorded. This is added to the manuscript’s method section in a new paragraph “adverse events”.**
