# Supplementary material for: Protocol for an international multicenter randomized controlled trial assessing treatment success and safety of peroral endoscopic myotomy vs endoscopic balloon dilation for the treatment of achalasia in children
Source: PLoS One. 2023 Oct 5;18(10):e0286880. doi: 10.1371/journal.pone.0286880 (PMC10553306; doi:10.1371/journal.pone.0286880)
Supplement: Attachment — Submitted filename: Review.docx [file pone.0286880.s005.docx]

Dear Editor：

Sorry for the delay of my review.

This is a multicentered randomized controlled trial comparing POEM AND pneumatic dilation for pediatric achalasia. There are several concerned aspects:

1. I am not sure whether a protocol without any trial results could be publised. There was no result addressing the efficacy or safty of POEM or dilation.
2. In page 4, line 88, “esophageal spasm” is present in type 3 achalasia.
3. In Introduction, mediacation such as nitrites is missing. Medication is an optional therapy.
4. In page 6,line 158. “Patients were not involved” in the study design. But in line 160, “21 pediatric patients” were involved. This is weired. Please clarify this.
5. In page 13, line 363, peumothorax is more frequent than pneumoperitium in POEM. Please check this.
6. In page 14, line 368, there was not necessary in discussing GERD in Methods.
7. Threre was no results in the article.
8. The discussion is too simple to draw a conclusion.
